# Multiple interactions of the dynein-2 complex with the IFT-B complex are required for effective intraflagellar transport

**DOI:** 10.1242/jcs.260462

**Published:** 2023-02-07

**Authors:** Shunya Hiyamizu, Hantian Qiu, Laura Vuolo, Nicola L. Stevenson, Caroline Shak, Kate J. Heesom, Yuki Hamada, Yuta Tsurumi, Shuhei Chiba, Yohei Katoh, David J. Stephens, Kazuhisa Nakayama

**Affiliations:** ^1^ Department of Physiological Chemistry, Graduate School of Pharmaceutical Sciences, Kyoto University, Sakyo-ku, Kyoto 606-8501, Japan; ^2^ Cell Biology Laboratories, School of Biochemistry, Faculty of Life Sciences, University of Bristol, Bristol BS8 1TD, UK; ^3^ Proteomics Facility, Faculty of Life Sciences, University of Bristol, Bristol BS8 1TD, UK; ^4^ Department of Genetic Disease Research, Graduate School of Medicine, Osaka City University, Abeno-ku, Osaka 545-8585, Japan

**Keywords:** Cilia, Dynein-2, IFT-B complex, Intraflagellar transport

## Abstract

The dynein-2 complex must be transported anterogradely within cilia to then drive retrograde trafficking of the intraflagellar transport (IFT) machinery containing IFT-A and IFT-B complexes. Here, we screened for potential interactions between the dynein-2 and IFT-B complexes and found multiple interactions among the dynein-2 and IFT-B subunits. In particular, WDR60 (also known as DYNC2I1) and the DYNC2H1–DYNC2LI1 dimer from dynein-2, and IFT54 (also known as TRAF3IP1) and IFT57 from IFT-B contribute to the dynein-2–IFT-B interactions. WDR60 interacts with IFT54 via a conserved region N-terminal to its light chain-binding regions. Expression of the WDR60 constructs in *WDR60*-knockout (KO) cells revealed that N-terminal truncation mutants lacking the IFT54-binding site fail to rescue abnormal phenotypes of *WDR60*-KO cells, such as aberrant accumulation of the IFT machinery around the ciliary tip and on the distal side of the transition zone. However, a WDR60 construct specifically lacking just the IFT54-binding site substantially restored the ciliary defects. In line with the current docking model of dynein-2 with the anterograde IFT trains, these results indicate that extensive interactions involving multiple subunits from the dynein-2 and IFT-B complexes participate in their connection.

## Teaser

**Summary:** The dynein-2 complex, a motor for retrograde protein trafficking within cilia, must be transported anterogradely as a cargo via extensive interactions of the dynein-2 complex with the IFT-B complex.

## INTRODUCTION

Primary cilia are antenna-like organelles that extend from the surface of various eukaryotic cell types. Cilia sense extracellular stimuli and receive and transduce developmental and homeotic signals, such as Hedgehog (Hh). To achieve these functions, there are specific proteins on the ciliary membrane, including G protein-coupled receptors (GPCRs) (Kopinke et al., 2021; Nachury and Mick, 2019). The ciliary membrane is separated from the plasma membrane, as the transition zone (TZ) located at the ciliary base serves as a diffusion and permeability barrier (Garcia-Gonzalo and Reiter, 2017). Owing to their crucial functions, defects in ciliogenesis and in ciliary protein trafficking lead to diverse hereditary disorders collectively referred to as the ciliopathies (Braun and Hildebrandt, 2017; Brown and Witman, 2014).

Bidirectional trafficking of ciliary proteins along the axonemal microtubules and import and export of proteins across the TZ are mediated by the intraflagellar transport (IFT) machinery, which was first identified in *Chlamydomonas* flagella but is well-conserved across eukaryotic species from *Chlamydomonas* to mammals (Prevo et al., 2017; Rosenbaum and Witman, 2002). The IFT machinery is a huge molecular complex containing the IFT-A, IFT-B and BBSome complexes (Nakayama and Katoh, 2020; Taschner and Lorentzen, 2016). In mammalian cilia, anterograde trafficking of the IFT machinery from the ciliary base to the tip is mediated by the IFT-B complex together with heterotrimeric kinesin-II (Funabashi et al., 2018; Kozminski et al., 1995), whereas the IFT-A complex, with the aid of the dynein-2 motor, plays a major role in retrograde trafficking (Vuolo et al., 2020; Webb et al., 2020). The IFT-A complex also mediates import of membrane proteins, including GPCRs, across the TZ together with the TULP3 adaptor protein (Badgandi et al., 2017; Hirano et al., 2017; Kobayashi et al., 2021; Mukhopadhyay et al., 2010). The hetero-octameric BBSome complex regulates dynein-2-driven retrograde trafficking and export of ciliary membrane proteins across the TZ by connecting IFT-B to the membrane proteins (Liu and Lechtreck, 2018; Nozaki et al., 2019, 2018; Yang et al., 2020; Ye et al., 2018).

**Fig. 1.**
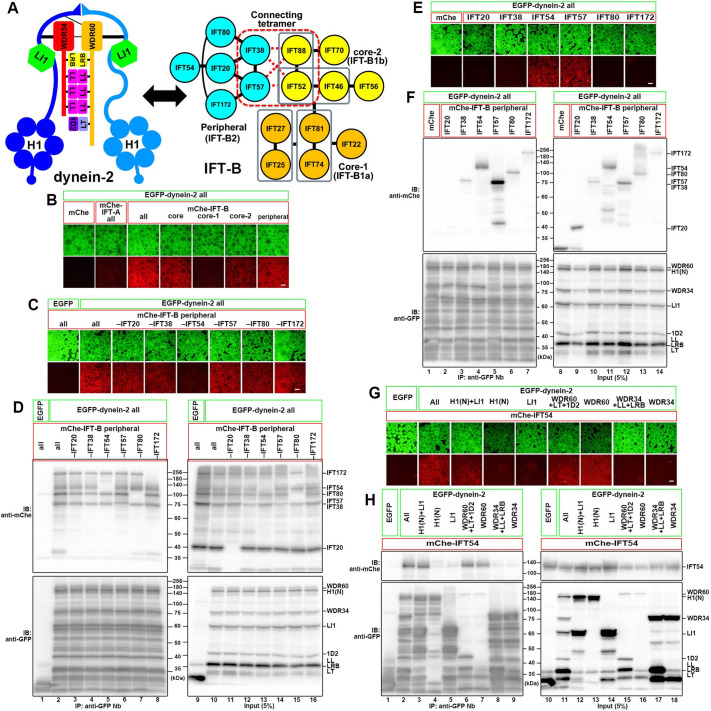
**Determination of subunits involved in the IFT-B–dynein-2 interactions.** (A) Schematic representation of the architectures of the dynein-2 and IFT-B complexes. (B) Dynein-2–IFT-B interaction revealed by the VIP assay. Lysates prepared from HEK293 T cells coexpressing all the dynein-2 subunits fused to EGFP and indicated IFT-A or IFT-B subunits fused to mCherry (mChe) were immunoprecipitated with GST-tagged anti-GFP Nb prebound to glutathione–Sepharose beads and subjected to the VIP assay. (C,D) Subtractive VIP assay and immunoblotting analysis to determine subunits of the IFT-B2 subcomplex required for its interaction with dynein-2. Lysates from cells coexpressing all the dynein-2 subunits fused to EGFP and all but one (as indicated) subunits of the IFT-B2 subcomplex fused to mChe were processed for the VIP assay (C) followed by immunoblotting analysis using anti-mChe and anti-GFP antibodies (D). (E,F) Determination of IFT-B2 subunits required for the interaction with dynein-2. Lysates from cells coexpressing all the dynein-2 subunits fused to EGFP and the indicated IFT-B2 subunit fused to mChe were processed for the VIP assay (E) followed by immunoblotting analysis (F). (G,H) Determination of dynein-2 subunits required for the interaction with IFT54. Lysates from cells coexpressing the indicated dynein-2 subunit(s) fused to EGFP and mChe-IFT54 were processed for the VIP assay (E) followed by immunoblotting analysis (F). Scale bars: 100 μm. 1D2, TCTEX1D2; H1, DYNC2H1; H1(N), DYNC2H1(N); LI1, DYNC2LI1; LL, DYNLL1 and DYNLL2; LRB, DYNLRB1 and DYNLRB2; LT, DYNLT1 and DYNLT3; IB, immunoblot; IP, immunoprecipitation. Images shown are representative of at least two repeats.

The IFT-A complex is composed of six subunits, with which TULP3 is associated (Hirano et al., 2017; Mukhopadhyay et al., 2010). By contrast, the IFT-B complex is composed of 16 subunits, which can be divided into two subcomplexes; the core (IFT-B1) subcomplex composed of 10 subunits [IFT22, IFT25 (HSPB11), IFT27, IFT46, IFT52, IFT56 (TTC26), IFT70 (IFT70A and IFT70B, also known as TTC30A and TTC30B, respectively), IFT74, IFT81 and IFT88] and the peripheral (IFT-B2) subcomplex composed of six subunits [IFT20, IFT38 (CLUAP1), IFT54 (TRAF3IP1), IFT57, IFT80 and IFT172]. These two subcomplexes are connected by composite interactions involving two IFT-B1 and two IFT-B2 subunits (IFT38, IFT52, IFT57 and IFT88) (Fig. 1A, right) (Katoh et al., 2016; Taschner et al., 2016).

Dynein-2, also known as IFT dynein, itself is also a multisubunit complex and is assembled around two copies of the motor subunit DYNC2H1 (Asante et al., 2014; Vuolo et al., 2020; Webb et al., 2020). In the human dynein-2 structure revealed by cryoelectron microscopy (cryo-EM), the light intermediate chain DYNC2LI1 binds to the N-terminal nonmotor tail region of each DYNC2H1 molecule (Toropova et al., 2017, 2019). The two DYNC2H1 tail regions adopt highly asymmetric conformations and associate mainly with the WD40 repeat domains of distinct intermediate chains, WDR60 and WDR34 (recently renamed as DYNC2I1 and DYNC2I2, respectively), which are heterodimerized via interacting with an array of the dimerized light chains [homo- or hetero-dimers of DYNLL1 and/or DYNLL2, homo- or hetero-dimers of DYNLRB1 and/or DYNLRB2, and heterodimers of either DYNLT1 or DYNLT3 and TCTEX1D2 (recently renamed as DYNLT2B); hereafter referred to as DYNLL, DYNLRB and DYNLT–TCTEX1D2 dimers, respectively] (Fig. 1A, left) (Hamada et al., 2018; Toropova et al., 2019; Tsurumi et al., 2019). It is noteworthy that mutations in all of the dynein-2-specific subunits and in all of the IFT-A subunits are known to cause skeletal ciliopathies of varying clinical severity (McInerney-Leo et al., 2015; Reiter and Leroux, 2017; Schmidts, 2014; Zhang et al., 2018). We recently revealed the relationships between defects in protein–protein interactions and ciliary defects caused by mutations of DYNC2LI1 and IFT-A subunits (IFT122 and IFT144) found in skeletal ciliopathies (Ishida et al., 2021; Qiu et al., 2022; Takahara et al., 2018).

Although the dynein-2 complex serves as a retrograde motor, it must be transported anterogradely as an IFT cargo (Iomini et al., 2001; Pedersen et al., 2006) while avoiding taking part in a ‘tug-of-war’ with kinesin-II. Docking of the human dynein-2 structure (Toropova et al., 2019) into the anterograde IFT train structure of *Chlamydomonas* flagella revealed by cryoelectron tomography (cryo-ET) (Jordan et al., 2018) and a recent cryo-ET study of assembling anterograde trains at the base of *Chlamydomonas* flagella (van den Hoek et al., 2022) has suggested that there are extensive contacts of the dynein-2 complex with the anterograde IFT trains (Jordan and Pigino, 2021; Webb et al., 2020); each dynein-2 complex is predicted to span out multiple IFT-B repeats by adopting an inactive conformation when it is transported as a cargo in an anterograde direction. Furthermore, a recent study using a combination of cryo-ET and AlphaFold2 structure predictions suggested that the dynein-2 complex primarily contacts the IFT-B2 side of the IFT-B unit repeat in the *Chlamydomonas* anterograde train (Lacey et al., 2023). However, details of the interactions involved in loading of the dynein-2 complex onto the IFT trains have not been investigated.

We showed previously that several of the IFT-B subunits in addition to the dynein-2 subunits are co-precipitated with HA-tagged WDR60 and/or WDR34 (Vuolo et al., 2018). In this study, we extended the analysis to find specific interactions between the dynein-2 and IFT-B complexes. In line with the docking model, suggesting extensive contacts of the dynein-2 complex with multiple IFT-B repeats in the anterograde trains, the data presented here indicate that multiple subunits from the dynein-2 and IFT-B complexes participate in their connection.

## RESULTS

### Reproducible co-precipitation of IFT-B2 subunits with WDR60 and WDR34

We previously used nano-liquid chromatography tandem mass spectrometry (nano-LC MS/MS) analysis of immunoprecipitates from lysates of RPE1 cells stably expressing HA-tagged WDR60 and WDR34 (Vuolo et al., 2018) to define the integrity of the dynein-2 complex following genome editing. Here, we expanded that work to robustly define IFT proteins that could be detected in such experiments including both HA and GFP tagging. As summarized in Table 1, we reproducibly detected interactions with IFT-B peripheral (IFT-B2) subcomplex proteins, IFT172, IFT54 and IFT57. IFT-B2 subunits IFT54 and IFT57 were found in all WDR60 proteomes, whereas IFT172 was found in six of seven WDR60 proteomes. Interactions were less robust with tagged WDR34 but, as with WDR60, IFT54 and IFT57 were the most reliably detected subunits. We also detected several of the core (IFT-B1) subunits (notably IFT88 and IFT70A) in most pull-down experiments with tagged WDR60. These results indicate that the dynein-2 complex binds to the IFT-B complex via direct or indirect interactions of WDR60 and/or WDR34 mainly with the IFT-B2 subunits. By contrast, interactions of IFT-A subunits with tagged WDR60 and WDR34 were not consistently detected, with IFT121 (also known as WDR35) being the most reproducibly identified. These results are consistent with a previous *Chlamydomonas* study indicating that the IFT-B complex can associate with dynein-2 independently of IFT-A (Pedersen et al., 2006) and with the recent proposed molecular model of the *Chlamydomonas* anterograde IFT train, in which the dynein-2 complex contacts primarily with the IFT-B2 side of the IFT-B unit repeat (Lacey et al., 2023).

**Table 1.**
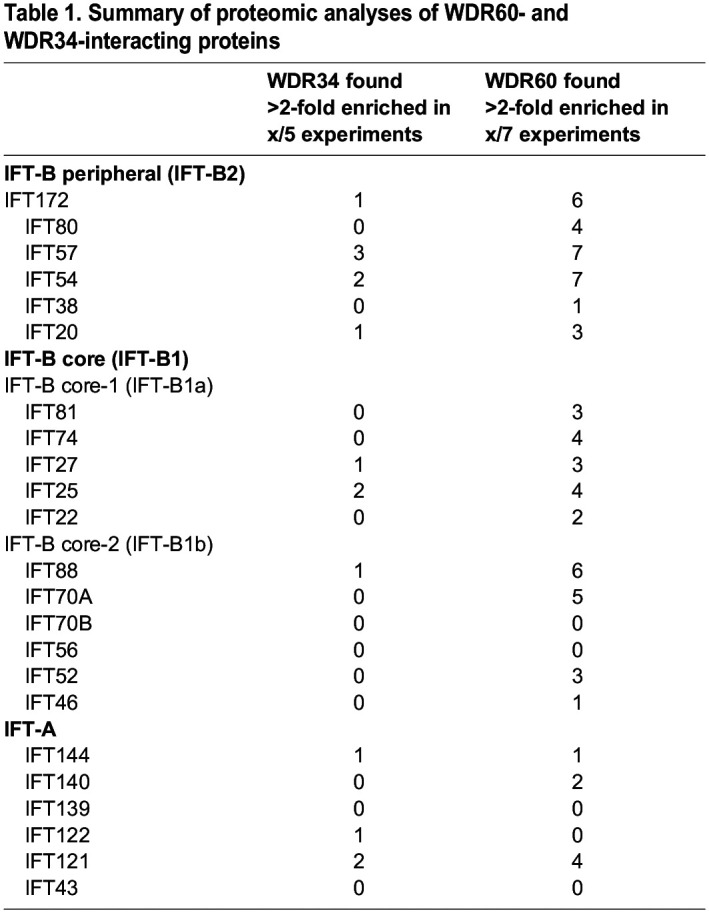
Summary of proteomic analyses of WDR60- and WDR34-interacting proteins

### Firm interactions of WDR60 and DYNC2H1–DYNC2LI1 with IFT-B2 subunits

To characterize these interactions in more detail, we systematically analyzed the interactions between the dynein-2 and IFT-B complexes by utilizing the visible immunoprecipitation (VIP) assay. The VIP assay is a versatile co-immunoprecipitation assay using fluorescent fusion proteins where not only binary but also one-to-many and many-to-many protein interactions can be visually detected (Katoh et al., 2015, 2016); however, it is important to note that the expression levels and stability of individual proteins could vary from protein to protein and be affected by co-expressed proteins and that the interactions could be affected by the fluorescent protein tags. In addition, ‘not detected’ in the VIP assay does not necessarily mean ‘no interaction’, as is true for many such interaction analyses using exogenously expressed proteins.

Lysates of HEK293T cells co-expressing all the dynein-2 subunits fused to EGFP and all the IFT-A or IFT-B subunits fused to mCherry were subjected to the VIP assay using glutathione S-transferase (GST)-tagged anti-GFP nanobody (Nb) prebound to glutathione–Sepharose beads. mCherry-fused IFT-B, but not IFT-A, demonstrated substantial red signals on the precipitated beads, indicating interaction(s) between the dynein-2 and IFT-B subunits (Fig. 1B, columns 2 and 3). When the IFT-B subunits were divided into the IFT-B1 and IFT-B2 subunits (Fig. 1A, right), IFT-B2 subunits demonstrated substantial interaction with the dynein-2 subunits (Fig. 1B, column 7) although the IFT-B1 subunits also demonstrated moderate interaction (column 4). When the IFT-B1 subunits were divided into the core-1 (IFT-B1a) and core-2 (IFT-B1b) subgroups, the later demonstrated interaction with dynein-2 subunits (column 6). These results suggest intricate and extensive interactions between the dynein-2 and IFT-B complexes. In view of the above proteomic analysis of WDR60- and WDR34-interacting proteins (Table 1) and the VIP assay results, we hereafter focused on the IFT-B2 subunits.

We then applied the subtractive VIP assay to determine which subunits in the IFT-B2 subcomplex are important for its interaction with the dynein-2 complex. As shown in Fig. 1C, omitting mCherry–IFT54 from the IFT-B2 subunits substantially reduced the red signals, suggesting that IFT54 makes a major contribution to the interaction of the IFT-B2 subcomplex with the dynein-2 complex. Furthermore, when the precipitated beads bearing fluorescent fusion proteins were processed for SDS-PAGE and subsequent immunoblotting analysis using anti-mCherry antibody (Fig. 1D), bands of the IFT-B2 subunits, except for the omitted subunit, were detected even in the absence of one of the subunits. Regarding the apparent discrepancy between the subtractive VIP and immunoblotting data when mCherry–IFT54 was omitted (Fig. 1C,D, column and lane 5), we suspect that despite the reduction in the red fluorescence in the absence of mCherry–IFT54, the other subunits retained their abilities to bind to the dynein-2 complex, albeit somewhat weakly.

We next examined interactions of individual IFT-B2 subunits with the dynein-2 complex. When lysates of cells co-expressing all the dynein-2 subunits fused to EGFP and individual IFT-B2 subunits fused to mCherry were subjected to the VIP assay, we saw strong association of mCherry-IFT54 and mCherry–IFT57 with the precipitated beads (Fig. 1E, columns 4 and 5). When the beads were subsequently processed for immunoblotting analysis using anti-mCherry antibody, robust bands were detected for mCherry–IFT54 and mCherry–IFT57 (Fig. 1F, lanes 4 and 5). mCherry-fused IFT172, IFT80 and IFT38 also gave rise to relatively weak bands (lanes 3, 6 and 7); note that the expression level and/or stability of mCherry–IFT172 was relatively low (lane 14) probably due to its large size. The finding that multiple subunits of the IFT-B complex participate in its interaction with the dynein-2 complex is not surprising in view of the current model of the dynein-2 loading onto anterograde IFT trains, predicting that the large dynein-2 complex spans over multiple repeats of the IFT-B complex in the anterograde IFT trains (Jordan et al., 2018; Toropova et al., 2019; van den Hoek et al., 2022).

In view of the proteomic analysis data of WDR60- and WDR34-interacting proteins (Table 1), we used the VIP assay to further interrogate how dynein-2 subunits interact with IFT54 and IFT57. The cryo-EM structure of human dynein-2 (Toropova et al., 2019) in conjunction with our biochemical data (Hamada et al., 2018; Qiu et al., 2022; Tsurumi et al., 2019) showed that the N-terminal nonmotor tail region of DYNC2H1 binds directly to DYNC2LI1, two DYNC2H1 tail regions interact mainly with the WD40 repeat domains of WDR60 and WDR34, and dimerized DYNLL light chains interact with the N-terminal non-WD40 regions of WDR60 and WDR34 to clump these intermediate chains (see Fig. 1A, left). Of the EGFP-fused dynein-2 subunits, WDR60 demonstrated the most robust interaction with mCherry–IFT54 (Fig. 1G,H, column and lane 7), although the expression level of EGFP–WDR60 was relatively low (Fig. 1H, lane 16), probably due to the instability of the N-terminal disordered region (see below). The data indicate that WDR60 alone can interact with IFT54, although it remains possible that coexpressed DYNLT light chains might alter the strength of the WDR60–IFT54 interaction (Fig. 1G,H, columns and lanes 6 and 7). While our study was in progress, Zhu et al. reported direct interaction of *Chlamydomona*s D1bLIC (the homolog of DYNC2LI1) with IFT54 and proposed that the interaction is crucial for anterograde transport of IFT dynein (Zhu et al., 2021); we could also detect a weak interaction between EGFP–DYNC2LI1 and mCherry–IFT54 (Fig. 1G,H, column and lane 5). In striking contrast, a substantial amount of mCherry–IFT54 was co-precipitated with anti-GFP Nb when the DYNC2H1(N) construct (residues 1–1090; the nonmotor tail region) was co-expressed with EGFP–DYNC2LI1 (Fig. 1G,H, column and lane 3). Thus, it is likely that DYNC2LI1 efficiently interacts with IFT54 when it is complexed with DYNC2H1.

**Fig. 2.**
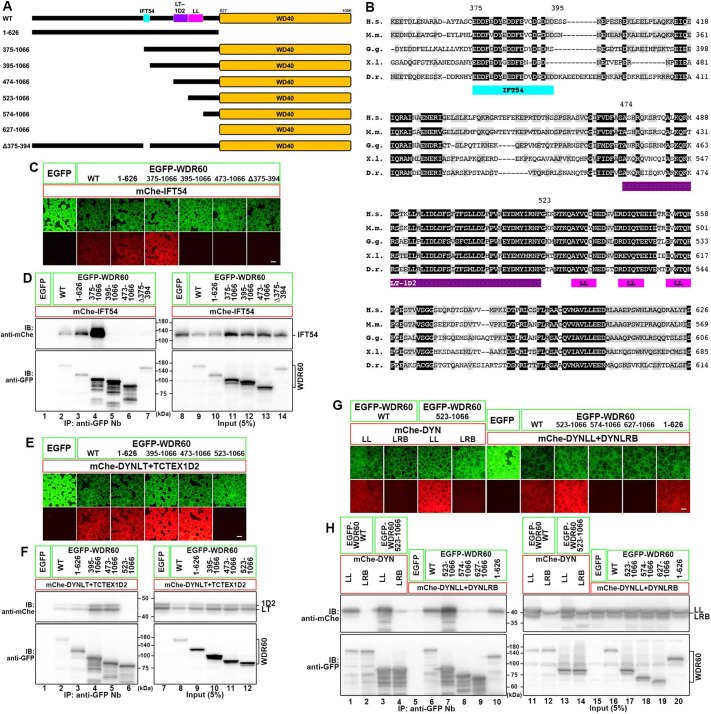
**WDR60 interacts with IFT54 via a region upstream of the light chain-binding sequences.** (A) WDR60 constructs used in this study. (B) Sequence alignment of the N-terminal region of vertebrate WDR60. Residues conserved in all species and those with conservative substitutions are in black and grey boxes, respectively. H.s., *Homo sapiens*; M.m., *Mus musculus*; G.g., *Gallus gallus*; X.l., *Xenopus laevis*; D.r., *Danio rerio*. In A and B, binding regions for IFT54, DYNLT–TCTEX1D2 and DYNLL are indicated. (C,D) Determination of the IFT54-binding region of WDR60. Lysates from cells coexpressing the indicated WDR60 construct fused to EGFP and mCherry (mChe)-IFT54 were processed for the VIP assay (C) followed by immunoblotting analysis (D). Note that expression levels of WDR60 constructs containing the N-terminal unstructured region were relatively low (lanes 9, 10 and 14). (E,F) Confirmation of the region of WDR60 required for its binding to the DYNLT–TCTEX1D2 dimer. Lysates from cells coexpressing the indicated WDR60 construct fused to EGFP and mChe-fused DYNLT1, DYNLT3 (DYNLT) and TCTEX1D2 were processed for the VIP assay (E) followed by immunoblotting analysis (F). (G,H) Confirmation of the region of WDR60 required for its binding to the DYNLL and DYNLRB dimers. Lysates from cells coexpressing the indicated WDR60 construct fused to EGFP and mChe-fused DYNLL1 and DYNLL2 (DYNLL) or DYNLRB1 and DYNLRB2 (DYNLRB) or both (DYNLL+DYNLRB) were processed for the VIP assay (G) followed by immunoblotting analysis (H). Scale bars: 100 μm. 1D2, TCTEX1D2; LL, DYNLL1 and DYNLL2; LRB, DYNLRB1 and DYNLRB2; LT, DYNLT1 and DYNLT3; IB, immunoblot; IP, immunoprecipitation. Images shown are representative of at least two repeats.

We also examined interactions of mCherry–IFT57 with EGFP-fused dynein-2 subunits by means of the VIP assay and subsequent immunoblotting analysis (Fig. S1A,B). We could detect robust interactions of mCherry–IFT57 with EGFP-fused DYNC2H1(N), DYNC2LI1, WDR60 and WDR34 (Fig. S1A,B, columns and lanes 4, 5, 7 and 9), indicating that IFT57 contacts multiple dynein-2 subunits. In view of the proteomic analysis data of WDR60-interacting proteins (Table 1), we then examined interactions of mCherry–IFT172 with dynein-2 subunits. Similar to what was found with IFT57, the results suggested interactions of IFT172 with multiple dynein-2 subunits, although the stability or expression level of the IFT172 protein was relatively low due to its large size (Fig. S1C,D). We also examined interactions of IFT38 or IFT80 with dynein-2 subunits as the data shown in Fig. 1F (lanes 3 and 6) suggested that these IFT-B2 subunits can directly, although weakly, interact with some dynein-2 subunits. The results shown in Fig. S1E–H suggest that IFT38 and IFT80 also interact with multiple dynein-2 subunits, in particular, with DYNC2LI1 (column and lane 5); in these cases, however, we could not clearly determine the interactions of IFT38 and IFT80 with WDR60, probably due to the instability of the WDR60 construct containing the N-terminal disordered region (Fig. S1F,H, lanes 15 and 16) as described above. Considering the proposed model where each dynein-2 complex spans across multiple IFT-B complexes, it is likely that these IFT-B2 subunits from multiple IFT-B units have contacts with distinct subunits or even different regions of the same subunit of a single dynein-2 complex. This is supported by the multiple interactions of IFT57 with WDR60. When the WDR60 protein was divided into the C-terminal WD40 repeat domain, WDR60(627–1066), and the N-terminal non-WD40 region, WDR60(1–626), we found that both WDR60 constructs were able to interact with IFT57 (Fig. S1I, columns 3 and 4). The most N-terminal non-conserved region, WDR60(1­–374) (see Fig. 2A,B), did not interact with IFT57 (Fig. S1I, column 5; summarized in Fig. S1J). On examination of other dynein-2 subunits, we found that WDR34 interacts with IFT57 via its C-terminal WD40 repeat domain (Fig. S1I, columns 7–9) and DYNC2LI1 interacts with IFT57 via its N-terminal dynein light intermediate chain domain (DLID) (Fig. S1I, columns 10–12; summarized in Fig. S1J).

### Interaction of WDR60 with IFT54 via its conserved region N-terminal of the light chain-binding regions

As the interactions of IFT57, IFT172, IFT38 and IFT80 with multiple dynein-2 subunits (Fig. S1A–H) hampered the detailed analysis of the interactions of these IFT-B subunits with dynein-2, we hereafter focused on the interaction between IFT54 and WDR60. WDR60 has a C-terminal WD40 repeat (β-propeller) domain and a residual N-terminal region (Fig. 2A), which is disordered in the dynein-2 cryo-EM structure (Toropova et al., 2019) and is relatively poorly conserved among species; in particular, the N-terminal 374-amino-acid region (for the human protein) is divergent even among vertebrate species (Fig. 2B). Using the VIP assay and the following immunoblotting analysis, both WDR60(1–626) and WDR60(375–1066) were found to interact with IFT54 (Fig. 2C,D, columns and lanes 3 and 4). Note that the expression levels of wild-type WDR60 [WDR60(WT)] and WDR60(1–626) were relatively low (Fig. 2D, lanes 9 and 10) and their band intensities detected by immunoblotting were weaker than expected from the corresponding signal intensities in the above VIP assay (Fig. 2C,D, columns and lanes 2 and 3; also see Fig. 2E,F, columns and lanes 2 and 3); this is probably due to the instability of the N-terminal intrinsically disordered region (Toropova et al., 2019). In striking contrast, a further 20-amino-acid truncation of the N-terminus of WDR60(375–1066) to WDR60(395–1066) abolished the interaction of WDR60 with IFT54 (Fig. 2C,D, column and lane 5). This 20-amino-acid region (residues 375–394) is highly conserved among vertebrates (Fig. 2B). The removal of the 20 amino acids from the full-length construct, WDR60(Δ375–394) (see Fig. 2A), also abolished the WDR60–IFT54 interaction (Fig. 2C,D, column and lane 7). Thus, the 20-amino-acid stretch (residues 375–394) of WDR60 is likely to be included in the IFT54-binding interface, although it remains possible that the 20-amino-acid deletion indirectly affects the overall protein structure.

We previously showed that WDR60 interacts with the light chain dimer DYNLT–TCTEX1D2 via residues 474–522 (see Fig. 2A,B) (Hamada et al., 2018). We confirm this here by showing that EGFP-fused WDR60(474–1066) but not WDR60(523–1066) coprecipitated a mixture of mCherry-fused DYNLT1, DYNLT3 and TCTEX1D2 (Fig. 2E,F, columns and lanes 5 and 6). Thus, the DYNLT-binding region appears not to overlap with the IFT54-binding region (Fig. 2A,B).

The cryo-EM structure of the human dynein-2 complex indicated that three DYNLL dimers interact with three short stretches located upstream of the C-terminal WD40 repeat domain of WDR60 and a DYNLRB dimer interacts with the region between the DYNLL-binding stretches and the WD40 repeats to bridge WDR60 and WDR34 (Toropova et al., 2019). However, our previous studies using the VIP assay detected the interactions of the DYNLL and DYNLRB dimers with WDR34 but not with WDR60 (Hamada et al., 2018; Tsurumi et al., 2019); we probably missed the interactions of WDR60 with these light chain dimers in the initial screening as we evaluated binary interactions as ‘positive’ only when red signals could be detected on the surface of the precipitated beads in reciprocal combinations of EGFP- and mCherry-fused proteins under fixed conditions (Hamada et al., 2018). We therefore reexamined whether WDR60 interacts with the DYNLL and DYNLRB dimers; in this study, we used a buffer solution for preparation of the cell lysates to increase the sensitivity of the VIP assay (Nishijima et al., 2017) compared to the original method (Katoh et al., 2015). As shown in Fig. 2G,H, a mixture of DYNLL1 and DYNLL2 bound robustly to WDR60(WT) and WDR60(523–1066) (columns and lanes 1 and 3). On the other hand, we could detect a very weak interaction of a mixture of DYNLRB1 and DYNLRB2 with WDR60(523–1066) (column and lane 4). When we examined whether a mixture of DYNLL1, DYNLL2, DYNLRB1 and DYNLRB2 interacts with WDR60 deletion constructs, WDR60(574–1066) had an extremely reduced ability to interact with the light chains as compared with WDR60(523–1066) (columns and lanes 7 and 8). The results are consistent with the cryo-EM study; the 51-amino-acid region (residues 523–573) contains three DYNLL-binding sequences (Toropova et al., 2019). Although we do not know the exact reason why the DYNLRB dimer bound to WDR60 at very low efficiency, the DYNLRB dimer might exhibit high-affinity binding to WDR60 when it simultaneously binds to WDR34 and WDR60 (Toropova et al., 2019), compared with what is seen in the absence of WDR34 as described previously (Vuolo et al., 2018).

### WDR60 and DYNC2H1–DYNC2LI1 bind to distinct regions of IFT54

**Fig. 3.**
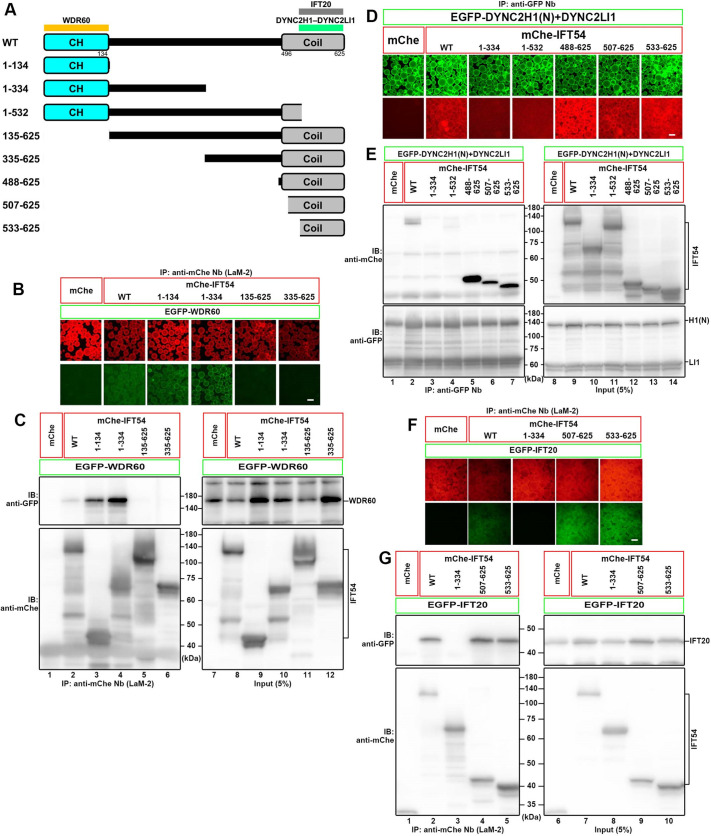
**WDR60 and DYNC2H1–DYNC2LI1 bind to distinct regions of IFT54.** (A) Structures of the IFT54 constructs. Binding regions for WDR60, DYNC2H1–DYNC2LI1 and IFT20 are indicated. CH, calponin homology; Coil, coiled-coil region. (B,C) Determination of the WDR60-binding region of IFT54. Lysates from cells coexpressing the indicated IFT54 construct fused to mCherry (mChe) and EGFP–WDR60 were processed for the VIP assay using GST-tagged anti-mChe Nb (LaM-2 version) (B) followed by immunoblotting analysis (C). (D,E) Determination of the binding region of IFT54 for DYNC2H1–DYNC2LI1. Lysates from cells coexpressing the indicated IFT54 construct fused to mChe and EGFP-fused DYNC2H1(N) plus DYNC2LI1 were processed for the VIP assay using GST-tagged anti-GFP Nb (D) followed by immunoblotting analysis (E). (F,G) Determination of the IFT20-binding region of IFT54. Lysates from cells coexpressing the indicated IFT54 construct fused to mChe and EGFP-IFT20 were processed for the VIP assay using GST-tagged anti-mChe Nb (F) followed by immunoblotting analysis (G). Scale bars: 100 μm. IB, immunoblot; IP, immunoprecipitation. Images shown are representative of at least two repeats.

We next determined the WDR60-binding region of IFT54. IFT54 has an N-terminal calponin-homology (CH) domain and C-terminal coiled-coil region (see Fig. 3A), which are connected by a flexible linker region. The coiled-coil region interacts with IFT20 in the IFT-B2 subcomplex (Katoh et al., 2016; Taschner et al., 2016; Petriman et al., 2022; Lacey et al., 2023). When the IFT54 protein was divided into two halves, the N-terminal region (residues 1–334) interacted with WDR60 (Fig. 3B,C, columns and lanes 4 and 6). The IFT54(1–134) construct retained the ability to interact with WDR60 (column and lane 3); note that for an unknown reason, the interaction with WDR60 of the full-length IFT54 construct was relatively weak compared with the C-terminal deletion constructs (Fig. 3C, compare lane 2 with lanes 3 and 4). By contrast, IFT54(135–625), which lacks the CH domain, did not interact with WDR60 (Fig. 3C, lane 5). Thus, the N-terminal CH domain is minimally required for IFT54 to interact with WDR60 (summarized in Fig. 3A).

We then determined the region of IFT54 responsible for its interaction with DYNC2H1–DYNC2LI1. In this case, the IFT54(488–625) construct, which covers the C-terminal coiled-coil region, could interact with DYNC2H1(N) plus DYNC2LI1 (Fig. 3D,E, column and lane 5), indicating that IFT54 interacts with WDR60 and DYNC2H1–DYNC2LI1 via distinct regions (see Fig. 3A). A further truncation analysis from the N-terminus revealed that IFT54(507–625) and IFT54(533–625) retained the ability to interact with DYNC2H1(N) plus DYNC2LI1 (columns and lanes 6 and 7); again, the interaction of full-length IFT54 with DYNC2H1(N) plus DYNC2LI1 was relatively weak compared with that of the N-terminal deletion constructs (Fig. 3E, compare lane 2 with lanes 5–7). By contrast, IFT54(1–532) did not interact with DYNC2H1(N) plus DYNC2LI1 (lane 4). Thus, the C-terminal coiled-coil region is important for the IFT54 interaction with DYNC2H1–DYNC2LI1. This is not entirely consistent with data from *Chlamydomonas* indicating that deletion of residues 261–275 of *Chlamydomonas* IFT54, which correspond to residues 393–407 of human IFT54 (see Fig. S2), weakened its interaction with D1bLIC (Zhu et al., 2021). The complex of DYNC2H1 and DYNC2LI1 might have extensive contacts with IFT54, compared with *Chlamydomonas* D1bLIC alone. As shown in our previous study (Katoh et al., 2016), and consistent with recent structural studies (Petriman et al., 2022; Lacey et al., 2023), IFT54 was confirmed to interact with IFT20 via its coiled-coil region, residues 533–625 (Fig. 3F,G, column and lane 5).

### WDR60 requires the N-terminal non-WD40 region for its normal function

**Fig. 4.**
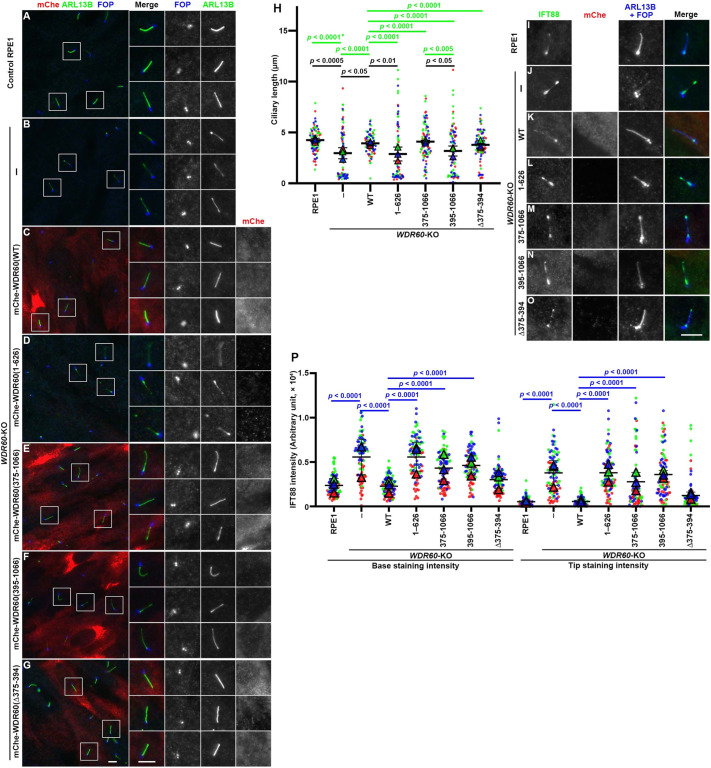
**The N-terminal and C-terminal regions of WDR60 are both required for normal trafficking of dynein-2.** (A–G,I–O) Control RPE1 cells (A,I), *WDR60*-KO cells (B,J) and those stably expressing mCherry (mChe)-fused WDR60(WT) (C,K), WDR60(1–626) (D,L), WDR60(375–1066) (E,M), WDR60(395–1066) (F,N) or WDR60(Δ375–394) (G,O) were serum-starved for 24 h and immunostained for ARL13B, RFP and FOP (recently renamed as CEP43) (A–G) or IFT88, RFP and ARL13B plus FOP (I–O). In A–G, boxed regions are 2.5-fold enlarged and shown on the right side. Scale bars: 5 µm. (H) Ciliary lengths of individual ciliated cells were measured and expressed as scatter plots. Differently colored dots represent three independent experiments (*n*=30×3), and triangles are means of individual experiments. Horizontal lines and error bars are the mean±s.d. of the three experiments. Statistical significances in the ciliary length (black lines and letters) and the ciliary length variation of individual cells (green lines and letters) were calculated using one-way ANOVA followed by the Tukey multiple comparison test and the *F* test, respectively. (P) The IFT88 staining intensities in the ciliary base and tip regions of individual ciliated cells were measured and expressed as scatter plots (*n*=30×3). Symbols are the same as in H. Statistical significances were calculated using one-way ANOVA followed by the Tukey test.

We then investigated whether the binding of dynein-2 to IFT-B via the WDR60–IFT54 interaction is required for the dynein-2 function. To this end, we analyzed phenotypes of *WDR60*-KO cells expressing the WDR60 constructs that were characterized in the experiments shown in Fig. 2. As described previously (Hamada et al., 2018), *WDR60*-KO cells had a more diverse ciliary length distribution than control RPE1 cells (Fig. 4A,B). In addition to the mean ciliary length (Fig. 4H, statistical significances are represented by *P*-values and lines shown in black), the length variability was eliminated through exogenous expression of mCherry-fused WDR60(WT) (Fig. 4C; also see Fig. 4H, statistical significances are represented by *P*-values and lines shown in green), although WDR60(WT) itself did not demonstrate distinct localization within cilia or at the ciliary base, as described previously (Hamada et al., 2018). By contrast, the WD40-lacking mutant WDR60(1–626), which lacks the ability to bind to DYNC2H1 (Hamada et al., 2018) and mimics one of the compound heterozygous alleles of a skeletal ciliopathy individual (McInerney-Leo et al., 2013; Vuolo et al., 2018), did not restore the normal cilia length distribution when expressed in *WDR60*-KO cells (Fig. 4D,H). Another abnormal phenotype of *WDR60*-KO cells is considerable enrichment of IFT-B components within cilia (Hamada et al., 2018; Vuolo et al., 2018); in *WDR60*-KO cells, IFT88 was significantly accumulated within cilia and the IFT88 accumulation was eliminated by expression of mCherry–WDR60(WT) (Fig. 4I–K). IFT88 was found to accumulate near the ciliary base as well as near the tip not only in *WDR60*-KO cells but also in those expressing WDR60(1–626) (Fig. 4J,L; also see Fig. 4P).

In *WDR60*-KO cells expressing mCherry-fused WDR60(375–1066) or WDR60(395–1066), the ciliary lengths of individual cells were more variable than in those expressing WDR60(WT) (Fig. 4E,F,H), and IFT88 was more enriched in both the base and tip regions than in those expressing WDR60(WT) (Fig. 4M,N,P). In *WDR60*-KO cells expressing mCherry-fused WDR60(Δ375–394), where the IFT54-binding site is specifically deleted (Fig. 2A–D), the ciliary length was significantly variable (Fig. 4G,H), although the base and tip level of IFT88 (Fig. 4O,P) were not significantly altered, compared with those expressing WDR60(WT). It is also notable that mCherry–WDR60(Δ375–394) tended to be faintly enriched near the ciliary base, albeit not in all cilia (Fig. 4G, enlarged mChe panels; also see Fig. 4O and Fig. 6G,U,BB); the relationship between the tendency of the enrichment and the variation in the ciliary length is so far unclear.

We also examined localization of IFT140, an IFT-A subunit, in *WDR60*-KO cells expressing one of mCherry–WDR60 constructs, as we have previously shown that the IFT-A complex is also significantly accumulated within *WDR60*-KO cilia (Fig. S3A) (Hamada et al., 2018). Stable expression of mCherry-fused WDR60(WT) (Fig. S3B), but not WDR60(1–626) (Fig. S3C), eliminated the IFT140 accumulation within cilia; in WDR60(WT)-expressing *WDR60*-KO cells, IFT140 was found predominantly at the ciliary base (Fig. S3B) like in control RPE1 cells (for example, see Hamada et al., 2018). In *WDR60*-KO cells expressing WDR60(375–1066), WDR60(395–1066) or WDR60(Δ375–394) (Fig. S3D–F), IFT140 was predominantly localized at the base like those expressing WDR60(WT). Thus, unlike in the case of IFT88, we could not find any clear difference among the WDR60 constructs, except for WDR60(1–626), concerning the IFT140 localization.

**Fig. 5.**
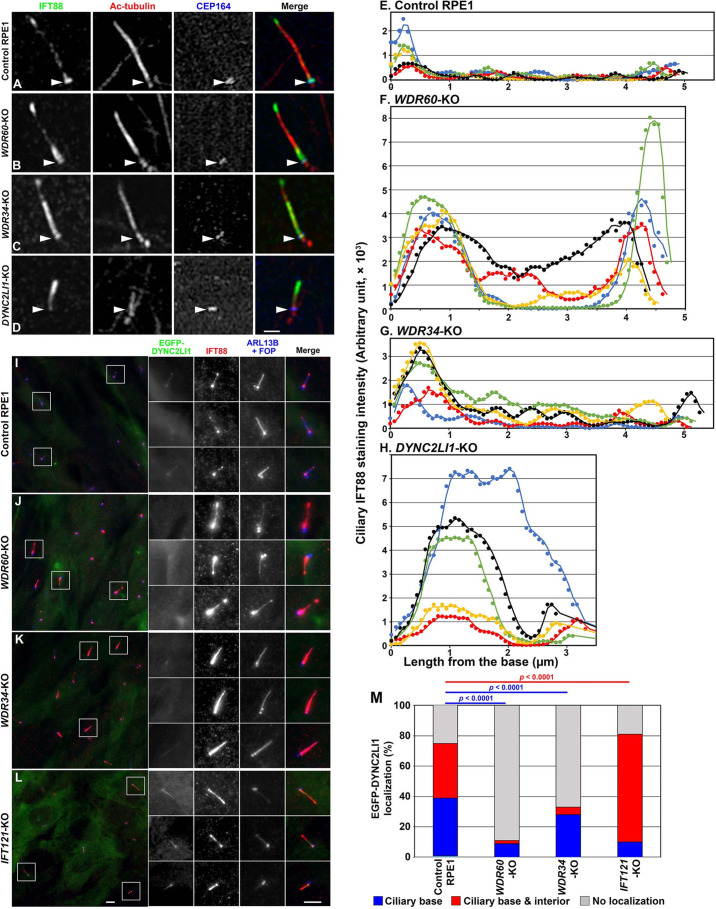
**IFT88 accumulation on the TZ distal side and at the ciliary tip and reduced DYNC2LI entry into cilia in *WDR60*-KO and *WDR34*-KO cells.** (A–D) Control RPE1 (A), *WDR60*-KO (B), *WDR34*-KO (C) and *DYNC2LI1*-KO (D) cells were serum-starved for 24 h and immunostained for IFT88, acetylated α-tubulin (Ac-tubulin) and CEP164. The stained cells were observed by Airyscan super-resolution microscopy. Arrowheads indicate the positions of CEP164-positive distal appendages. Scale bar: 1 µm. (E–H) Line scans of IFT88 staining intensities along individual cilia of control RPE1 (E), *WDR60*-KO (F), *WDR34*-KO (G) and *DYNC2LI1*-KO (H) cells. Line scans of cilia with lengths that fall within 10% of the mean length are shown (*n*=5). (I–L) Control RPE1 (I), *WDR60*-KO (J), *WDR34*-KO (K) and *IFT121*-KO (L) cells stably expressing EGFP–DYNC2LI1 were serum-starved for 24 h and immunostained for IFT88 and ARL13B plus FOP. Boxed regions are 2.5-fold enlarged and shown on the right side. Scale bars: 5 µm. (M) Localization of EGFP-DYNC2LI1 in control RPE1, *WDR60*-KO, *WDR34*-KO and *IFT121*-KO cells was classified as ‘ciliary base’, ‘ciliary base and interior’, and ‘no ciliary localization’. The cells in each population (in 100 ciliated cells analyzed) were counted and the percentages of these populations are represented as stacked bar graphs. Statistical significances were calculated using the Pearson χ^2^ test. Images shown are representative of two repeats.

While this study was under way, De-Castro et al. (2022) reported that in *Caenorhabditis elegans wdr-60* mutants (a null mutant and a mutant expressing a WD40-lacking WDR-60 protein), IFT components were accumulated within sensory cilia, in particular on the distal side of the TZ, probably due to reduced ciliary entry of the remaining dynein-2 complex in the absence of WDR60. Analysis of control RPE1 cells using Airyscan super-resolution microscopy revealed that IFT88 was predominantly found at the ciliary base (Fig. 5A), in particular, in the distal appendage region labeled with CEP164, as described previously (Ishida et al., 2021; Katoh et al., 2020). In line with the observations in *C. elegans wdr-60* mutants, IFT88 was substantially enriched in the region over the CEP164-positive distal appendages as well as around the ciliary tip in *WDR60*-KO cells (Fig. 5B). Line scanning of IFT88 staining images along *WDR60*-KO cilia acquired by conventional microscopy revealed a considerable enrichment of IFT88 more distal to the base (Fig. 5F) compared to control RPE1 cilia (Fig. 5E). Like *WDR60*-KO cells, *WDR34*-KO cells also demonstrated substantial enrichment of IFT88 on the distal side of the TZ and around the tip, with the former appearing to predominate (Fig. 5C,G). By contrast, in *DYNC2LI1*-KO cells, IFT88 was considerably enriched in a distal region within severely shortened cilia (Fig. 5D,H) (see Discussion), as described previously (Qiu et al., 2022).

To test the proposal of De-Castro et al. (2022) that, in the absence of WDR-60, ciliary entry of the incomplete dynein-2 complex is reduced, thereby causing IFT components to accumulate distally to the TZ, we compared the localization of stably expressed EGFP–DYNC2LI1 between control RPE1 and *WDR60*-KO cells. In control cells, EGFP–DYNC2LI1 was found within cilia and at the base (Fig. 5I) as described previously (Hamada et al., 2018). By contrast, the majority of *WDR60*-KO cells did not demonstrate EGFP–DYNC2LI1 signals within cilia or at the ciliary base (Fig. 5J; also see Fig. 5M). These observations indicate that the lack of WDR60 decreases the efficiency of assembly of the dynein-2 complex and/or decreases the entry of the incomplete dynein-2 complex into cilia, presumably by impairing its loading onto the anterograde IFT trains. Similar to what is seen in *WDR60*-KO cells, ciliary EGFP–DYNC2LI1 signals were significantly reduced in *WDR34*-KO cells compared to control RPE1 cells (Fig. 5K,M). In striking contrast, EGFP–DYNC2LI1 signals within cilia were significantly increased in *IFT121*-KO cells (Fig. 5L,M), in which ciliary retrograde trafficking is impaired due to incomplete IFT-A complex formation (Hirano et al., 2017; Takahara et al., 2018) but the dynein-2 complex itself is likely to be intact.

**Fig. 6.**
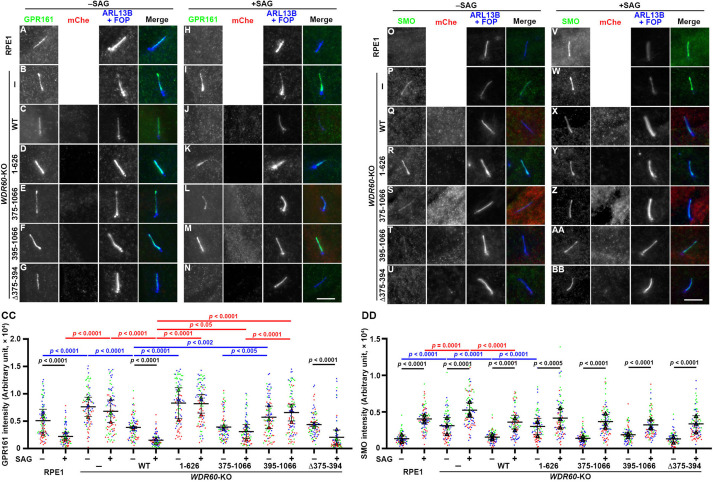
**Defects in induced export of GPR161 from cilia in *WDR60*-KO cells expressing WDR60 mutants.** (A–BB) Control RPE1 cells (A,H,O,V), *WDR60*-KO cells (B,I,P,W) and those stably expressing mCherry (mChe)-fused WDR60(WT) (C,J,Q,X), WDR60(1–626) (D,K,R,Y), WDR60(375–1066) (E,L,S,Z), WDR60(395–1066) (F,M,T,AA) or WDR60(Δ375–394) (G,N,U,BB) were serum-starved for 24 h and then incubated for a further 24 h in the absence (–SAG) or presence (+SAG) of 200 nM SAG. The cells were immunostained for either GPR161 (A–N) or SMO (O–BB), RFP and ARL13B plus FOP (A–BB). Scale bars: 5 µm. (CC,DD) The ciliary GPR161 and SMO staining intensities of individual ciliated cells were measured and expressed as scatter plots (*n*=30×3). Symbols used are the same as in Fig. 4H. Horizontal lines and error bars are the mean±s.d. Statistical significances were calculated using one-way ANOVA followed by the Tukey test for comparison among multiple samples and the unpaired two-tailed Student's *t*-test for comparison between –SAG and +SAG.

We next analyzed changes in the localization of GPR161 and Smoothened (SMO), in response to the Hh pathway activation, as retrograde trafficking and export from cilia of these GPCRs mediated by the IFT machinery and the BBSome are thought to be driven by dynein-2. These GPCRs participate in the Hh signaling pathway; in the basal state, GPR161 on the ciliary membrane negatively regulates the Hh signaling, whereas upon the Hh pathway activation, SMO enters and GPR161 exits cilia, resulting in the removal of negative regulation (Kopinke et al., 2021; Fig. 6A,O). When the Hh pathway is activated by treatment of cells with Smoothened Agonist (SAG), GPR161 exits cilia (Fig. 6H) whereas SMO enters cilia (Fig. 6V). In *WDR60*-KO cells, ciliary GPR161 and SMO levels are higher than those in control cells under basal conditions (Fig. 6B,P; also see Fig. 6CC,DD), suggesting that basal recycling of these GPCRs are suppressed in the absence of WDR60. The SAG-induced ciliary levels of GPR161 and SMO were also significantly higher in *WDR60*-KO cells than those in control cells (Fig. 6I,W,CC,DD). The GPR161 and SMO levels were restored to control levels by stable expression of mCherry–WDR60(WT) (Fig. 6C,J,Q,X,CC,DD). In striking contrast, stable expression of the WD40-lacking mutant WDR60(1–626) in *WDR60*-KO cells had little effect on the ciliary GPR161 and SMO levels, and the changes in these levels upon SAG treatment (Fig. 6D,K,R,Y,CC,DD). These observations indicate that retrograde trafficking and/or export of these GPCRs is impaired in the absence of WDR60.

*WDR60*-KO cells expressing mCherry–WDR60(375–1066) and those expressing mCherry–WDR60(395–1066) demonstrated slightly different phenotypes with respect to the ciliary GPR161 level. The basal ciliary GPR161 level was significantly high in *WDR60*-KO cells expressing WDR60(395–1066) (Fig. 6F), which lacks an IFT54-binding site, but not in those expressing WDR60(375–1066) (Fig. 6E), compared with those expressing mCherry–WDR60(WT) (Fig. 6CC). Upon treatment with SAG, the ciliary GPR161 level was not significantly decreased in both *WDR60*-KO cells expressing WDR60(375–1066) and WDR60(395–1066), unlike those expressing WDR60(WT) (Fig. 6L,M; also see Fig. 6CC). Thus, it is likely that SAG-induced export from cilia and/or retrograde trafficking of GPR161 is impaired in both *WDR60*-KO cells expressing WDR60(375–1066) and WDR60(395–1066) to varying degrees. By contrast, the basal and SAG-induced levels of SMO in WDR60(375–1066)-expressing and WDR60(395–1066)-expressing *WDR60*-KO cells were not significantly altered compared with WDR60(WT)-expressing *WDR60*-KO cells (Fig. 6S,T,Z,AA,DD). In *WDR60*-KO cells expressing mCherry-fused WDR60(Δ375–394), which specifically lacks the IFT54-binding site (Fig. 2A–D), the basal and SAG-stimulated levels of GPR161 and SMO were not significantly different from those in WDR60(WT)-expressing cells (Fig. 6G,N,U,BB–DD).

## DISCUSSION

In order to achieve its function as a retrograde IFT motor, the dynein-2 complex must be transported to the ciliary tip as an inactive cargo via binding to the IFT machinery (Vuolo et al., 2020; Webb et al., 2020). Consistent with this notion, our data show that when expressed in hTERT-RPE1 cells, HA-tagged WDR60 and WDR34 co-precipitate not only other dynein-2 subunits but also several subunits of the IFT-B complex, in particular, those of the IFT-B2 subcomplex (Vuolo et al., 2018). Our data show that both WDR60 and WDR34 reproducibly co-precipitate IFT-B2 subunits but inconsistently co-precipitate the IFT-A subunits (Table 1). This agrees well with the *Chlamydomonas* anterograde IFT train model assembled using a combination of cryo-ET and the AlphaFold2 predictions, in which the dynein-2 complex has extensive contacts with the IFT-B2 side of the IFT-B repeats (Lacey et al., 2023). The dynein-2 interaction with IFT-B2 is also supported by the AlphaFold model of the IFT-B complex validated using cross-linking and mass spectrometry analysis (Petriman et al., 2022). In other words, our biochemical data and the structural models, including predictions with AlphaFold2, complement each other, even though they have been presented independently. In view of the current docking model, in which the large dynein-2 complex has extensive contacts with multiple units of the IFT-B complex, but not directly with the IFT-A complex, in the anterograde IFT trains (Jordan and Pigino, 2021; Toropova et al., 2019), the anterograde train model (Lacey et al., 2023) and the cyto-ET structure of assembling IFT trains at the ciliary base (van den Hoek et al., 2022), the interactions of WDR60, WDR34 and DYNC2H1–DYNC2LI1 with multiple IFT-B subunits are likely to mainly represent those occurring when the dynein-2 complex is transported as an anterograde IFT cargo, although the possibility that these interactions also occur during retrograde trafficking cannot be completely excluded. There are technical reasons why some subunits might be more readily detected than others using this approach, but overall, these data provide strong evidence of robust interactions within the context of intact multiprotein complexes. The abundance of IFT-B proteins and the relative lack of IFT-A proteins might indicate weaker binding of IFT-A to dynein-2, lower abundance or a relative lack of stability of retrograde complexes.

Further analyses utilizing the VIP assay revealed a multitude of interactions between the dynein-2 and IFT-B complexes, as predicted from the models in which the dynein-2 complex has extensive contacts with multiple IFT-B repeats, but not directly with the IFT-A complex, in the anterograde IFT trains (Toropova et al., 2019; Petriman et al., 2022; Lacey et al., 2023). In agreement with the proteomics data, IFT54, IFT57 and IFT172 of the IFT-B2 subcomplex were found to make major contributions to the IFT-B interactions with the dynein-2 complex, although other IFT-B subunits also participate in the interaction with dynein-2 (Fig. 1). These data show that IFT54 interacts with both WDR60 and the DYNC2H1–DYNC2LI1 heterodimer, whereas IFT57 and IFT172 interact with all the dynein-2 subunit(s) examined – DYNC2H1, DYNC2LI1, WDR60 and WDR34. Subsequent analyses revealed that distinct regions of IFT54 interact with WDR60 and DYNC2H1–DYNC2LI1, namely the N-terminal CH domain and the C-terminal coiled-coil region, respectively (Fig. 2). These multiple contacts between the dynein-2 and IFT-B complexes are in agreement with the docking model where each dynein-2 complex spans out multiple IFT-B repeats of the anterograde train (Toropova et al., 2019). By contrast, WDR60 interacts with IFT54 via a conserved region (residues 375–394) that is N-terminal to the light chain-binding regions, as well as with IFT57, and probably IFT172, via distinct regions (Fig. S1). The WDR60 N-terminal region is likely to be flexible to interact with other molecules, as the region is disordered in the cryo-EM structure (Toropova et al., 2019).

Consistent with a recent study on *C. elegans wdr-60* mutants (De-Castro et al., 2022), *WDR60*-KO cells established from RPE1 cells and those expressing WDR60(1–626), which lacks the WD40 repeats and cannot bind DYNC2H1, demonstrated aberrant accumulation of IFT88 on the distal side of the TZ as well as around the ciliary tip (Fig. 4J,L,P; Fig. 5B,F). Although WDR60 or WDR34 is not required for ciliogenesis (Hamada et al., 2018; Tsurumi et al., 2019; Vuolo et al., 2018), we showed recently that only very short cilia are formed in the absence of DYNC2LI1 (Qiu et al., 2022), which stabilizes the motor subunit DYNC2H1 (Toropova et al., 2019). This is consistent with previous data (Vuolo et al., 2018) and those presented in this study (Fig. 5I–M) showing that loss of WDR60 or WDR34 results in reduced assembly or reduced entry into cilia of the dynein-2 complex. Our interaction data (Fig. 2) suggest that the dynein-2 complex containing DYNC2H1–DYNC2LI1, but lacking WDR60 or containing truncated WDR60, has a weaker interaction with the IFT-B complex, thereby making it less likely to enter cilia via loading onto anterograde IFT trains; this is supported by our proteomics data showing robust pull-down of IFT-B proteins with WDR60 (Table 1). The reduced dynein-2 loading onto IFT trains could result in a reduction in retrograde trafficking from the ciliary tip and in passage of the IFT machinery through the TZ (De-Castro et al., 2022; Park and Leroux, 2022). In contrast to *WDR60*-KO and *WDR34*-KO cells, *DYNC2LI1*-KO cells accumulated IFT88 in the distal region of short cilia, but not on the distal side of the TZ (Fig. 5A–H). In the same vein, *C. elegans wdr-60* mutant accumulates the IFT components around the ciliary tip and on the distal side of the TZ (De-Castro et al., 2022), whereas *xbx-1* (DYNC2LI1) and *che-3* (DYNC2H1) mutants do not demonstrate enrichment of the IFT components on the TZ distal side but do accumulate them in the distal region of shortened cilia (Jensen et al., 2018; Schafer et al., 2003). By contrast, TZ integrity is compromised in *xbx-1* and *che-3* mutants but not in *wdr-60* mutants (De-Castro et al., 2022; Jensen et al., 2018). This contrasts with the situation in mammalian cells where knockout of WDR60 also affects TZ integrity (Vuolo et al., 2018). Thus, it is likely that in the absence of WDR60 or WDR34, dynein-2 is loaded onto the anterograde trains to some extent and drives retrograde trafficking and passage through the TZ of the IFT machinery with reduced kinetics as proposed by De-Castro et al. (2022), whereas loss of DYNC2H1 or DYNC2LI1 makes dynein-2 non-functional and somehow more severely compromises the TZ integrity.

*WDR60*-KO cells expressing WDR60(375–1066), which lacks the N-terminal non-conserved region but retains both the IFT54-binding site and the ability to hold the dimerized light chains together with WDR34, show moderate defects in retrograde trafficking, in passage through the TZ of the IFT machinery and in induced exit of GPR161 from cilia. Thus, the non-conserved region (residues 1–374) of WDR60 might also contribute to the dynein-2 function. Although we could not yet detect any proteins that interact with the non-conserved region, a cryo-EM study of the dynein-2 complex suggested that the flexible N-terminal region of WDR60 is located within potential contact distance to the IFT-B complex (Jordan et al., 2018; Toropova et al., 2019). The AlphaFold2 Database also predicts that the N-terminal region is largely unstructured (Tunyasuvunakool et al., 2021). Furthermore, as the region is unstable (Fig. 2D, lanes 9 and 10), we might be unable to detect proteins interacting with it. Therefore, it remains possible that as ‘an intrinsically disordered region’, the N-terminal region of WDR60 is involved in interactions with IFT-B subunits including IFT54.

*WDR60*-KO cells expressing WDR60(395–1066), which lacks the IFT54-binding site as well as the N-terminal non-conserved region, exhibited slightly but significantly more severe defects than those expressing WDR60(375–1066) (see Figs 4H and 6CC). This suggests that binding of WDR60 to IFT54 and other IFT-B subunits participates in the function of the dynein-2 complex, probably by contributing to its transport as an IFT cargo. The pivotal role of IFT54 in loading of the dynein-2 complex onto IFT trains is supported by two *Chlamydomonas* studies. A recent careful observation of *Chlamydomonas* IFT trains by cryo-ET indicated that expression of fluorescent protein-tagged IFT54 subtly alters the anterograde train structure causing a reduction in both binding of IFT dynein and in its import into flagella (Wingfield et al., 2021). Expression of an IFT54 construct with weakened D1bLIC binding in the *Chlamydomonas ift54*-null mutant decreases the ciliary level and the ciliary entry frequency of IFT dynein (Zhu et al., 2021). However, *WDR60*-KO cells expressing WDR60(Δ375–394), which specifically lacks the IFT54-binding site, exhibited nearly normal phenotypes, with subtle differences, compared to those expressing WDR60(WT). This was somewhat unexpected if the WDR60–IFT54 interaction is crucial for the dynein-2 trafficking mediated by the IFT trains. Given that the individual dynein-2 subunits WDR60, WDR34, DYNC2LI1 and probably DYNC2H1, within a single dynein-2 complex can have multiple contacts with multiple IFT-B subunits from multiple IFT-B repeats in the IFT trains (Jordan et al., 2018; Toropova et al., 2019; Webb et al., 2020; Lacey et al., 2023), the loss of only one of the multiple interactions between the dynein-2 and IFT-B complexes might only marginally affect the strength of the overall interaction. For example, the largest subunit DYNC2H1 probably makes substantial contributions to the dynein-2 loading onto the anterograde trains, as the *Chlamydomonas* anterograde train model suggested that both the motor domain and the nonmotor tail region of DYNC2H1 have multiple contacts with multiple IFT-B2 units (Lacey et al., 2023), although our interaction analyses using the entire region or the motor domain of DYNC2H1 have so far been unsuccessful due to its extremely large size.

This study did not reveal or characterize all the interactions among components of the dynein-2 complex and those of the IFT machinery, leaving some unexplained issues concerning abnormal phenotypes of *WDR60*-KO cells expressing WDR60 mutants. Furthermore, this study does not reveal the interactions of the dynein-2 complex with the IFT machinery that are involved in driving retrograde trafficking, nor does it completely rule out the possibility that the interactions that have been detected are involved in retrograde trafficking. In order to understand the mechanisms underlying not only anterograde trafficking of the dynein-2 complex but also retrograde trafficking driven by dynein-2, which involve such a large number of and intricate interactions, we must continue to steadily elucidate the interactions and will have to wait for detailed structural analysis in the future. For this purpose, cross-linking and mass spectrometry (Piersimoni et al., 2022) might be powerful in elucidating intricate interaction patterns between large protein complexes.

Multiple ciliopathies caused by mutations in *DYNC2H1*, *WDR60*, *WDR34*, *DYNC2LI1* and *TCTEX1D2* have been reported (McInerney-Leo et al., 2015; Schmidts et al., 2015; Zhang et al., 2018). These mutations are likely to affect important ciliary functions, such as Hh signaling, by impairing retrograde trafficking and export of ciliary proteins. Our approach to elucidating the relationships between mutation-induced changes in protein–protein interactions and abnormalities at the cellular level will continue to contribute to the understanding of the pathogenesis of ciliopathies.

## MATERIALS AND METHODS

### Plasmids, antibodies, reagents and cell lines

Constructs of dynein-2 and IFT-B subunits used in this study are listed in Table S1. Other constructs were described previously (Hamada et al., 2018; Katoh et al., 2016; Qiu et al., 2022; Tsurumi et al., 2019). Antibodies used in this study are listed in Table S2. Glutathione S-transferase (GST)-tagged anti-GFP Nb and anti-mCherry Nb (LaM-2 version) prebound to glutathione–Sepharose 4B beads were prepared as described previously (Ishida et al., 2021; Katoh et al., 2015). SAG was purchased from Enzo Life Sciences. hTERT-RPE1 and HEK293 T cells were obtained from American Type Culture Collection (CRL-4000) and RIKEN BioResource Research Center (RBC2202), respectively. *WDR60*-KO (the #W60-2-2 cell line), *WDR34*-KO (#W34-1-5), *DYNC2LI1*-KO (#LI1-3-2) and *IFT121*-KO (#121-1-3) cells were established from hTERT-RPE1 cells as described previously (Hamada et al., 2018; Qiu et al., 2022; Takahara et al., 2018; Tsurumi et al., 2019).

### Immunoprecipitation and proteomic analysis

hTERT-RPE1 cells expressing the indicated cDNA constructs were washed with phosphate-buffered saline (PBS) and incubated with crosslinker solution [1 mM dithiobis(succinimidyl propionate), Thermo Fisher Scientific #22585] for 30 min on ice. The reaction was quenched by adding 500 mM Tris-HCl pH 7.5 for 15 min. Immunoprecipitation of lysates of hTERT-RPE1 cells stably expressing HA-tagged or GFP-tagged WDR60 or WDR34 was performed using anti-HA agarose beads (Sigma-Aldrich) or GFP-Trap agarose beads (Chromotek).

Lysis buffer containing 50 mM Tris-HCl pH 7.4, 1 mM EDTA, 150 mM NaCl, 1% Igepal (CA-630, MP Biomedicals, 198596) and protease inhibitors (539137, Millipore) was used for HA immunoprecipitation and a buffer of 10 mM Tris-HCl pH 7.4, 50 mM NaCl, 0.5 mM EDTA, protease inhibitors and 0.5% Igepal was used for GFP immunoprecipitation. Subsequently, cells were incubated on a rotor at 4°C for 30 min and then lysates were centrifuged at 13,000 ***g*** at 4°C for 10 min. Cell lysates were added to the equilibrated HA or GFP beads and incubated on a rotor at 4°C. Next, the anti-HA beads were washed in washing buffer containing 50 mM Tris-HCl pH 7.4, 150 mM NaCl, 0.5 mM EDTA, 0.3% Triton X-100, 0.1% SDS and GFP-Trap beads were washed in a buffer of 10 mM Tris-HCl pH 7.4, 50 mM NaCl and 0.5 mM EDTA. Subsequent proteomic analysis by nano-LC MS/MS using an Orbitrap Fusion Tribrid mass spectrometer (Thermo Fisher Scientific) was performed as described previously (Vuolo et al., 2018).

The raw data were processed using Proteome Discoverer software v2.1 (Thermo Fisher Scientific) and searched against the UniProt Human database and (where relevant) GFP sequence using the SEQUEST algorithm. Peptide precursor mass tolerance was set at 10 ppm, and MS/MS tolerance was set at 0.6 Da. Search criteria included oxidation of methionine (+15.9949) as a variable modification and carbamidomethylation of cysteine (+57.0214) and (where used in the original experiment) the addition of the TMT mass tag (+229.163) to peptide N-termini and lysine as fixed modifications. Searches were performed with full tryptic digestion and a maximum of one missed cleavage was allowed. The reverse database search option was enabled, and the data was filtered to satisfy a false discovery rate of 5%.

For TMT experiments, the resulting Peptide Abundance Ratios from TMT experiments were obtained by taking the ratio of peptide abundance (for example) using HA–WDR60 divided by that of HA–GFP. We chose 2-fold enrichment as an arbitrary cut-off for enrichment based on detection of known components of the dynein-2 complex and other known interactions while eliminating detection of known contaminants. For samples analyzed using GFP-Trap, peptide abundances were calculated as a ratio of peptides detected with GFP–WDR34 or GFP–WDR60 divided by those detected with GFP alone.

The mass spectrometry proteomics data have been deposited to the ProteomeXchange Consortium via the PRIDE partner repository (Perez-Riverol et al., 2022) with the dataset identifiers PXD031151, PXD031152, PXD031153, PXD031154, PXD031156, PXD031157 and PXD031158.

### VIP assay and immunoblotting analysis

VIP assays were performed as described previously (Katoh et al., 2018, 2015), with minor modifications (Ishida et al., 2021; Nishijima et al., 2017). Lysates were prepared from HEK293 T cells transfected with expression vectors for EGFP-fused and mCherry-fused proteins using HMDEKN cell lysis buffer (10 mM HEPES, pH 7.4, 5 mM MgSO_4_, 1 mM dithiothreitol, 0.5 mM EDTA, 25 mM KCl, 0.05% NP-40). After immunoprecipitation with GST-tagged anti-GFP Nb or anti-mCherry Nb (LaM-2) prebound to glutathione–Sepharose 4B beads, the beads bearing the fluorescent fusion proteins were observed under a fluorescence microscope (BZ-8000, KEYENCE). The beads were then boiled in SDS-PAGE sample buffer and subjected to SDS-PAGE and immunoblotting analysis using anti-GFP and anti-mCherry antibodies as described previously (Katoh et al., 2015, 2016). Full blot images for blots shown in this paper are presented in Fig. S4.

### Preparation of WDR60-KO cells stably expressing the mCherry-fused WDR60 construct

Lentiviral vectors for mCherry-fused WDR60 constructs were prepared as described previously (Hamada et al., 2018; Takahashi et al., 2012). HEK293 T cells were transfected with pRRLsinPPT-mCherry–WDR60 or its deletion construct together with the packaging plasmids [pRSV-REV, pMD2.g and pMDLg/pRRE; kind gifts from Peter McPherson, McGill University, Montreal, Canada (Thomas et al., 2009)]. The culture medium was replaced 8 h after transfection. Culture media containing lentiviral particles were collected at 24, 36 and 48 h after transfection, passed through a 0.45-µm filter and centrifuged at 32,000 ***g*** at 4°C for 4 h. Precipitated viral particles were resuspended in Opti-MEM (Thermo Fisher Scientific) and stored at −80°C until use. *WDR60*-KO cells expressing the mCherry-fused WDR60 construct were prepared by the addition of the lentiviral suspension to the culture medium and processed for immunofluorescence analysis.

### Immunofluorescence analysis

hTERT-RPE1 cells were cultured in DMEM/F-12 (Nacalai Tesque) supplemented with 10% fetal bovine serum and 0.348% sodium bicarbonate. To induce ciliogenesis, cells were grown to 100% confluence on coverslips and serum-starved for 24 h in DMEM/F-12 containing 0.2% bovine serum albumin.

Immunofluorescence analysis was performed as described previously (Nakamura et al., 2020; Nozaki et al., 2017; Zhou et al., 2022). Cells were fixed and permeabilized with 3% paraformaldehyde at 37°C for 5 min and subsequently in 100% methanol for 5 min at −20°C, and washed three times witj PBS (for experiments shown in Figs 4, 5, 6O–BB, Fig. S3), or were fixed with 3% paraformaldehyde at 37°C for 15 min, washed two times with PBS, quenched with 50 mM NH_4_Cl at room temperature for 15 min, washed once with PBS, subsequently permeabilized with PBS containing 0.1% Triton X-100 at room temperature for 5 min and washed three times with PBS (for experiments shown in Fig. 6A–N). The fixed and permeabilized cells were blocked with 10% fetal bovine serum, stained with antibodies diluted in 5% fetal bovine serum (for experiments shown in Figs 4, 5, 6A–N), or stained with antibodies diluted in Can Get Signal Immunostain Solution A (Toyobo) (for experiments shown in Fig. 6O–BB). The immunostained cells were observed using an Axio Observer microscope (Carl Zeiss). Quantification was performed as described previously (Qiu et al., 2022). Briefly, all images acquired under the same setting and saved in CZI file format were processed and analyzed using the ZEN3.1 microscope software (Carl Zeiss). A new model of cilia was created by drawing the contour of cilia along the signal of ARL13B in object channel using the Intellesis trainable segmentation module of ZEN. After training many times, the model in the Intellesis trainable segmentation could automatically recognize most cilia. After manually excluding regions that were incorrectly identified as cilia, the Image Analysis application was able to use the model to automeasure ciliary length. A region of interest (ROI) was created by drawing a line along the signal of ARL13B within cilia using a Draw Spline Contour tool in the ZEN 3.1 imaging software, and the fluorescence intensity in the ROI was quantified. To measure fluorescence intensity at the tip and base of cilia, ROIs were created by drawing a circle at the tip and base of cilia using a Draw Circle tool in the ZEN 3.1 imaging software. To correct for local background intensity, the ROIs were duplicated and set to a nearby region. Statistical analyses were performed using GraphPad Prism8 (Version 8.4.3; GraphPad Software). Airyscan super-resolution imaging was performed using the LSM800 microscope (Carl Zeiss) at the Research Support Platform in Osaka City University as described previously (Katoh et al., 2020; Okazaki et al., 2020). To measure IFT88 staining intensities along individual cilia, ROIs were created by drawing a curve along cilia from the base (position of FOP staining) to the tip using a Draw Curve tool in the ZEN 3.1 imaging software, and the fluorescence intensity in the ROI was quantified. To correct for local background intensity, the ROI was duplicated and set in a nearby region.

## Supplementary Material

10.1242/joces.260462_sup1 Supplementary information
