# Supplementary material for: Multiple interactions of the dynein-2 complex with the IFT-B complex are required for effective intraflagellar transport
Source: J Cell Sci. 2023 Feb 7;136(5):jcs260462. doi: 10.1242/jcs.260462 (PMC10110421; doi:10.1242/jcs.260462)
Supplement: Supplementary information [file joces-136-260462-s1.pdf]

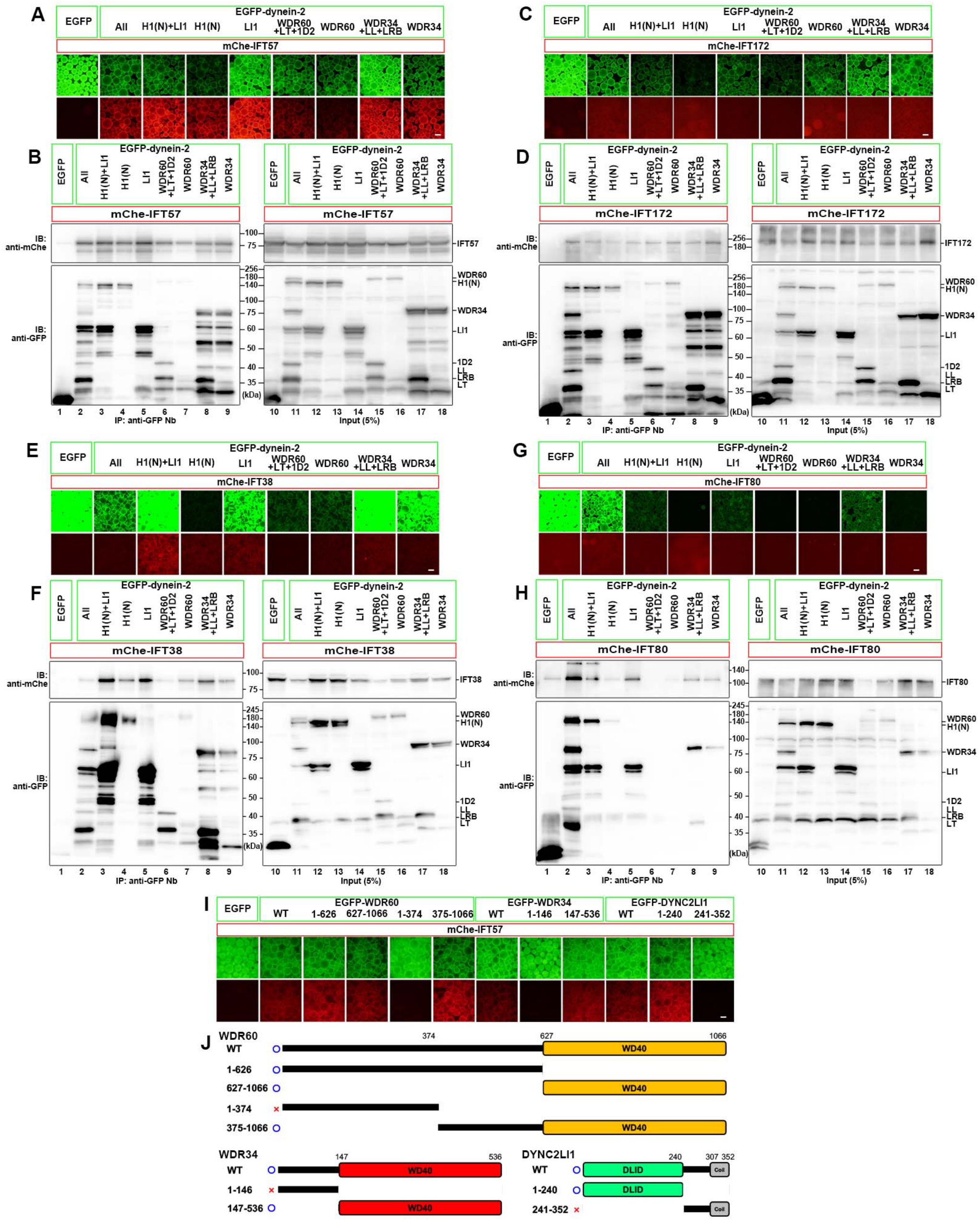

Fig. S1. Determination of the IFT57-binding regions of WDR60, WDR34, and DYNC2LI1

(A–H) VIP assay and immunoblotting analysis to determine subunits of the dynein-2 complex required for its interaction with IFT57 and IFT172. Lysates from cells coexpressing the indicated dynein-2 subunit(s) fused to EGFP and mChe-fused IFT57 (A, B), IFT172 (C, D), IFT38 (E, F), or IFT80 (G, H) were processed for the VIP assay (A, C, E, G) followed by immunoblotting analysis (B, D, F, H). Note that a weak non-specific band of unknown origin was detected in lane 1 as a negative control in Fig. 1H. (I) VIP assay to determine the IFT57-binding region of WDR60, WDR34, and DYNC2LI1. Lysates from cells coexpressing the indicated WDR60, WDR34, or DYNC2LI1 construct fused to EGFP and mChe-IFT57 were processed for the VIP assay. Scale bars: 100 µm. LL, DYNLL1 and DYNLL2; LRB, DYNLRB1 and DYNLRB2; LT, DYNLT1 and DYNLT3. (J) Schematic representation of the structures of WDR60, WDR34, and DYNC2LI1 constructs used in experiments shown in this figure.

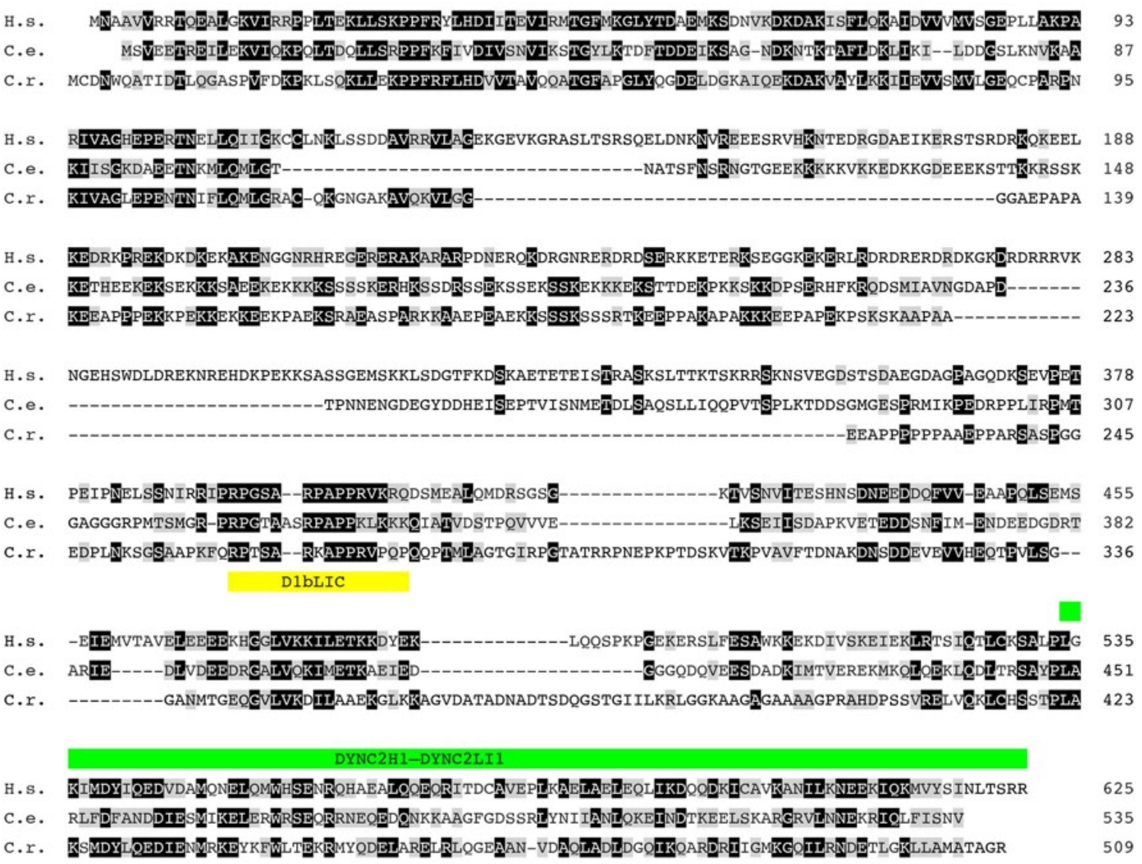

Fig. S2. IFT54 sequence alignment

Sequence alignment of *Homo sapiens* (H.s.), *Caenorhabditis elegans* (C.e.), and *Chlamydomonas reinhardtii* (C.r.) IFT54. Residues conserved at least between two species are shown in black boxes and those with conservative substitutions are in grey boxes. The binding region for *Chlamydomonas* D1bLIC predicted from the study of Zhu *et al.* (Zhu et al., 2021) and the DYNC2H1–DYNC2LI1-binding region determined in this study are indicated.

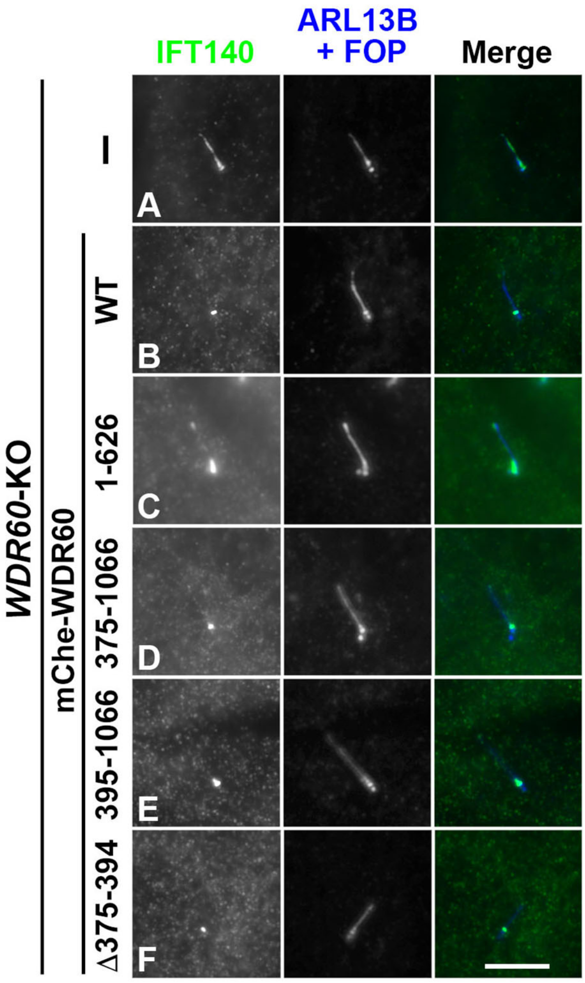

Fig. S3. Enrichment of IFT140 within cilia in *WDR60*-KO cells and in those expressing WDR60(1–626) *WDR60*-KO cells (A) and those stably expressing mCherry (mChe)-fused WDR60(WT) (B), WDR60(1–626) (C), WDR60(375–1066) (D), WDR60(395–1066) (E), or WDR60(Δ375–394) (F) were serum-starved for 24 h and immunostained for IFT140 and ARL13B +FOP. Scale bar, 5 µm. Note that the epifluorescence of mChe-fused proteins was not detectable without staining with anti-RFP antibody because the cells were fixed and permeabilized with cold methanol (see Materials and Methods).

Journal of Cell Science • Supplementary information

Fig. S4A: Uncropped images of Fig. 1D

Fig. 1D (upper left)

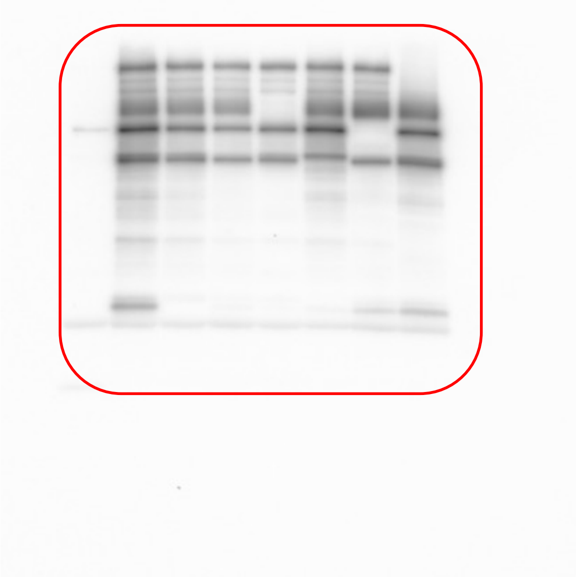

Fig. 1D (upper right)

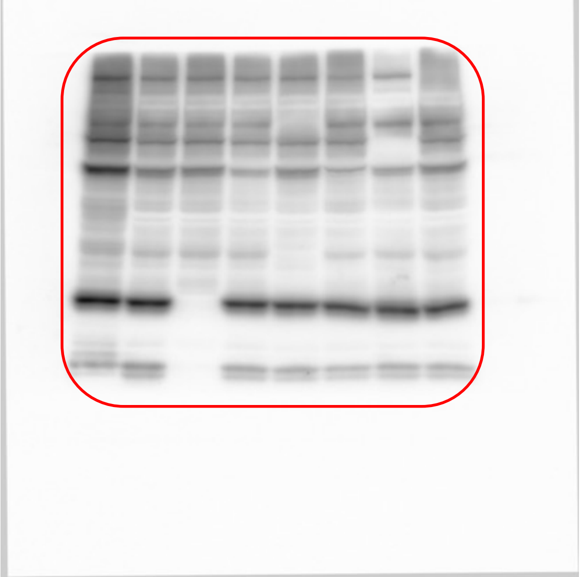

Fig. 1D (lower left)

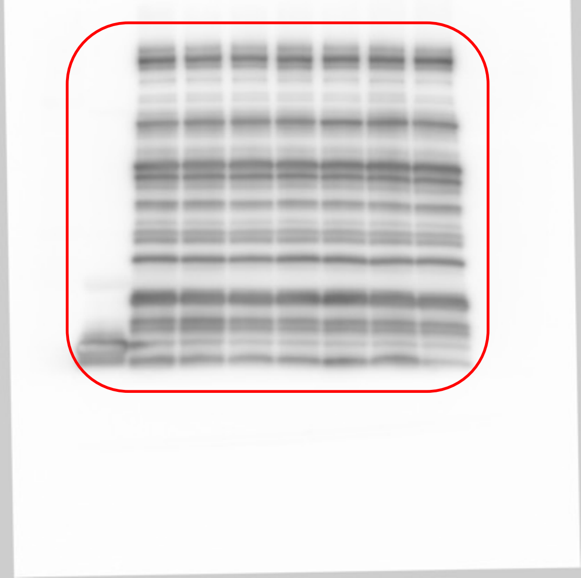

Fig. 1D (lower right)

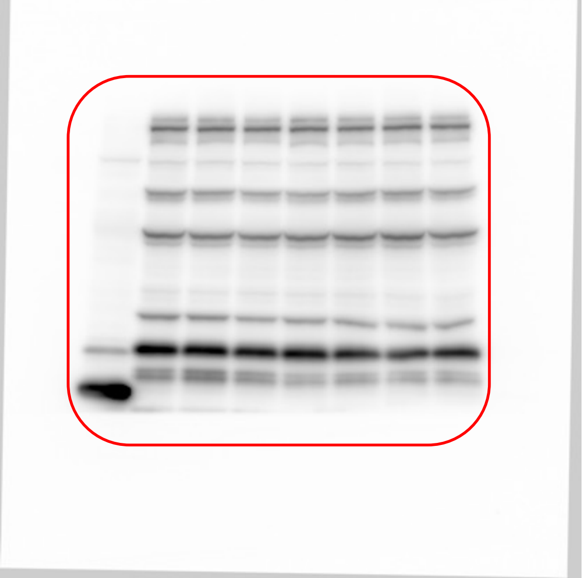

Fig. S4B: Uncropped images of Fig. 1F

Fig. 1F (upper left)

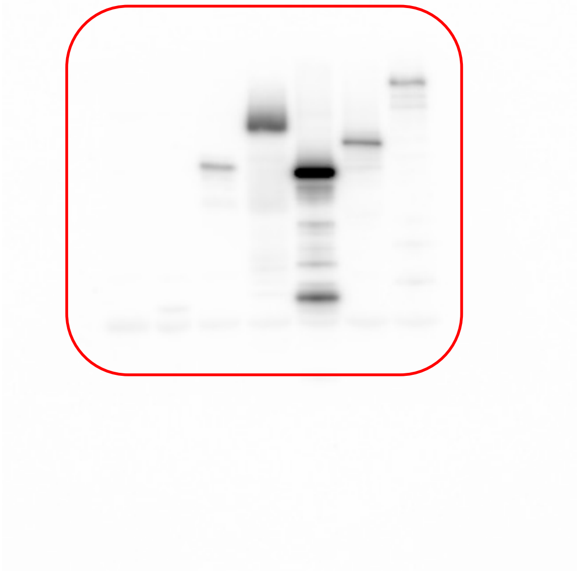

Fig. 1F (upper right)

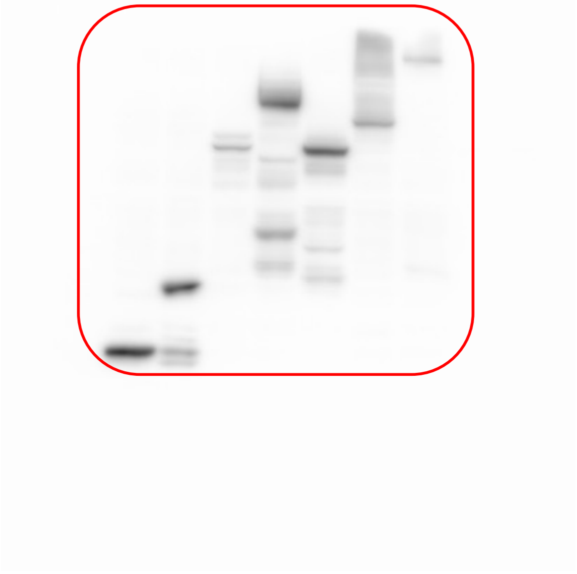

Fig. 1F (lower left)

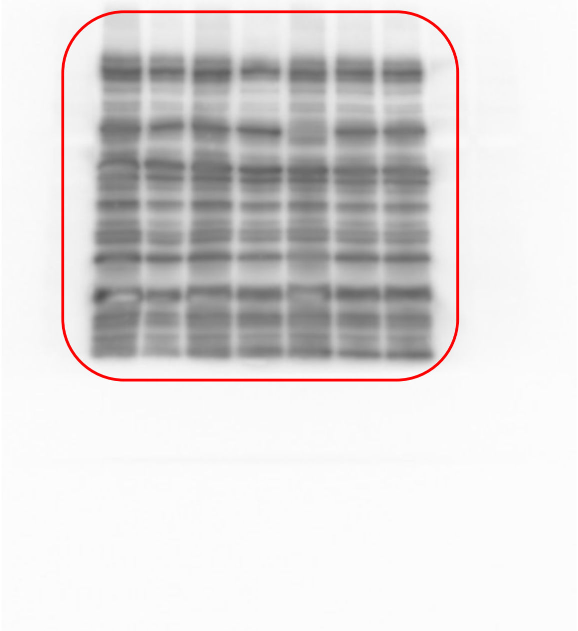

Fig. 1F (lower right)

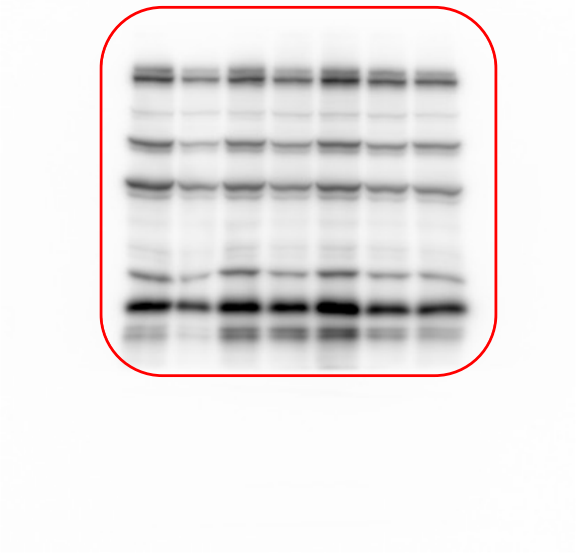

Journal of Cell Science • Supplementary information

Fig. S4C: Uncropped images of Fig. 1H

Fig. 1H (upper left)

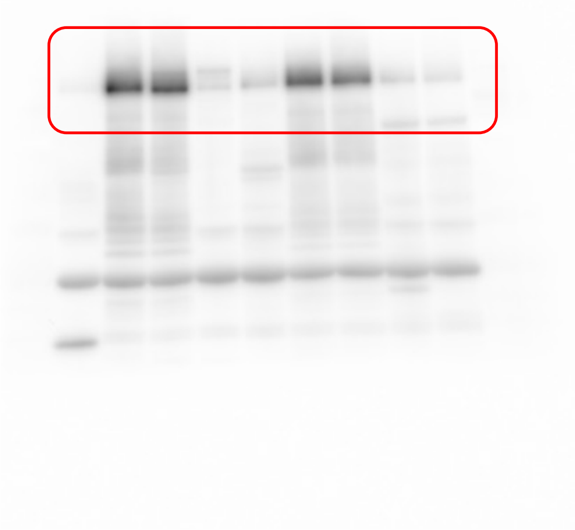

Fig. 1H(upper right)

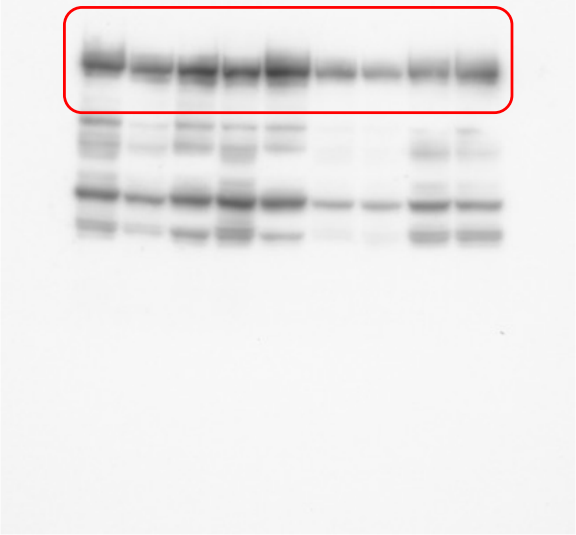

Fig. 1H (lower left)

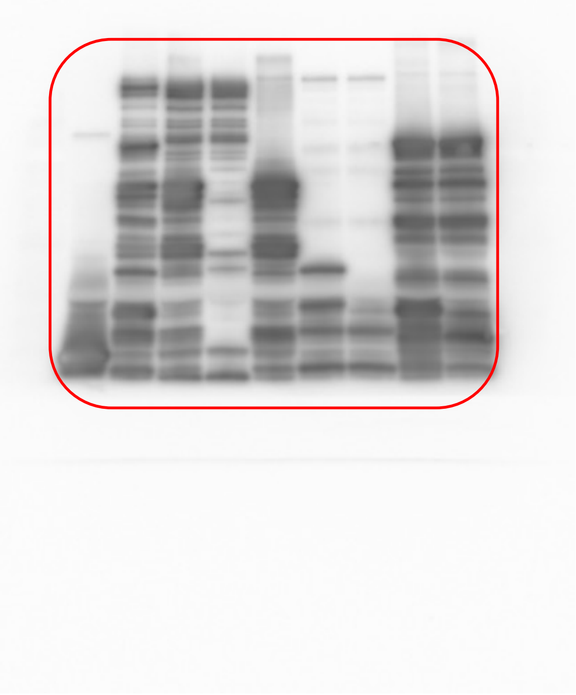

Fig. 1H (lower right)

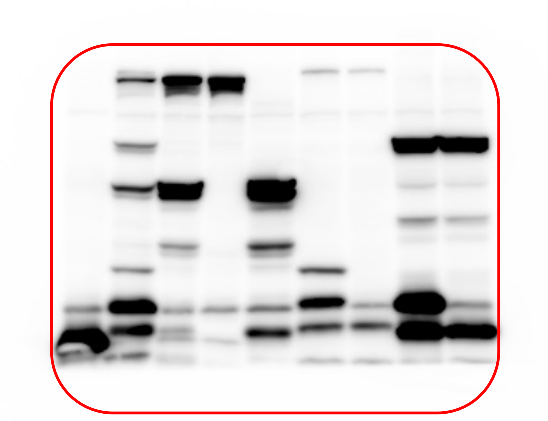

Journal of Cell Science • Supplementary information

Fig. S4D: Uncropped images of Fig. 2D

Fig. 2D (upper left)

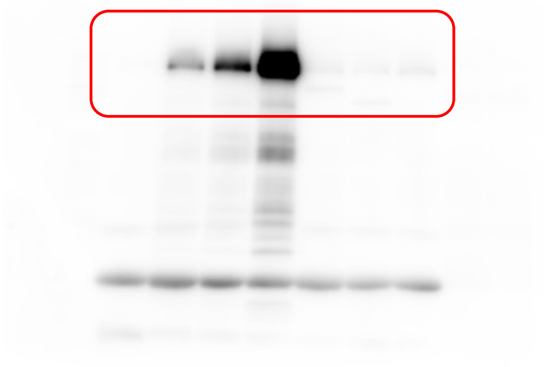

Fig. 2D (upper right)

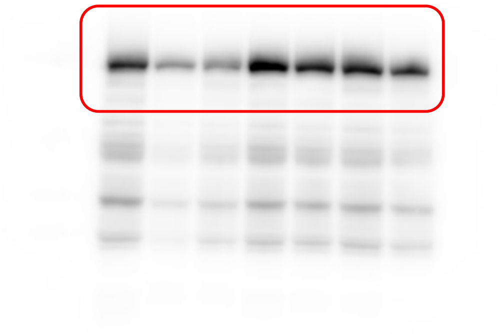

Fig. 2D (lower left)

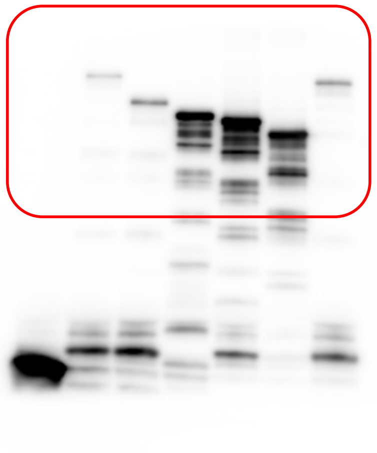

Fig. 2D (lower right)

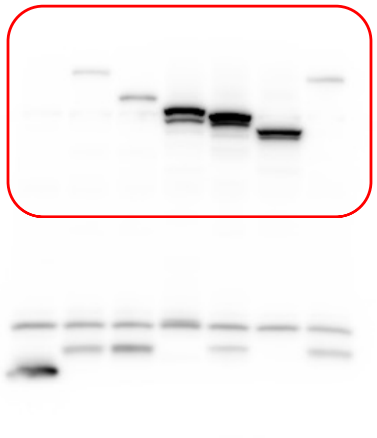

Fig. S4E: Uncropped images of Fig. 2F

Fig. 2F (upper left)

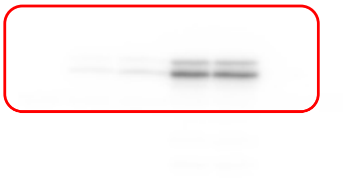

Fig. 2F (upper right)

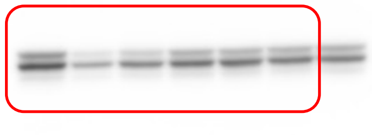

Fig. 2F (lower left)

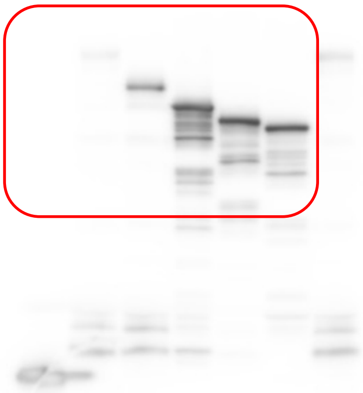

Fig. 2F (lower right)

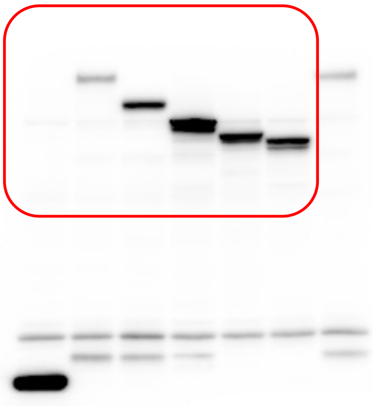

Fig. S4F: Uncropped images of Fig. 2H

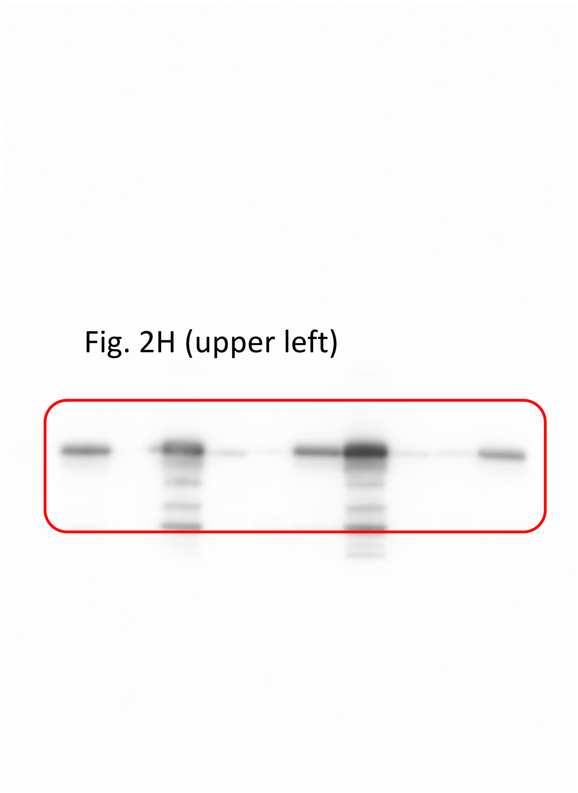

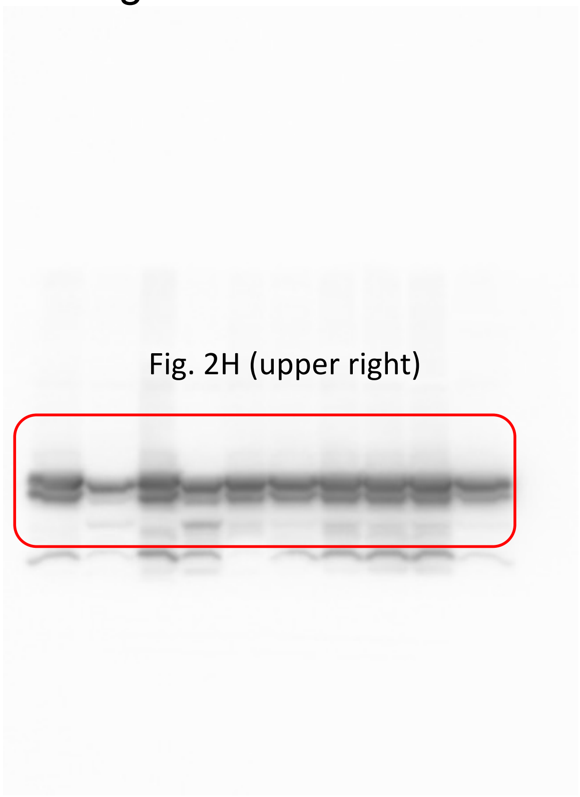

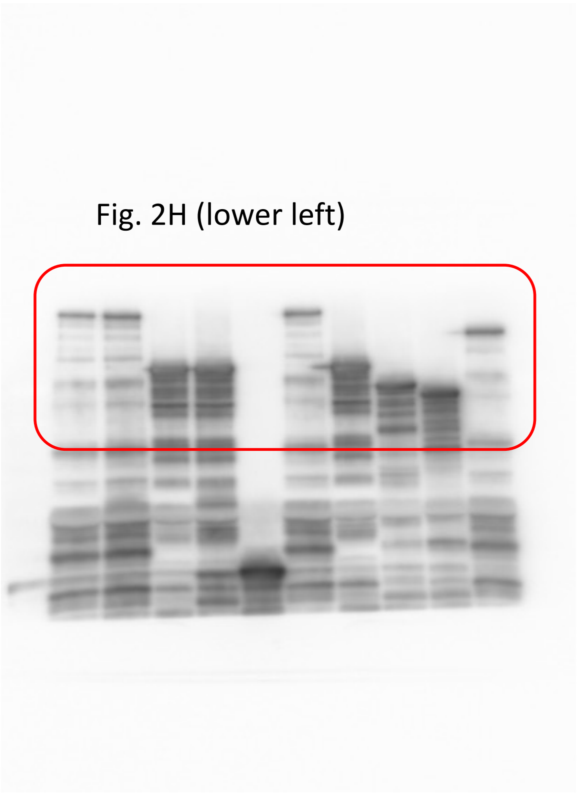

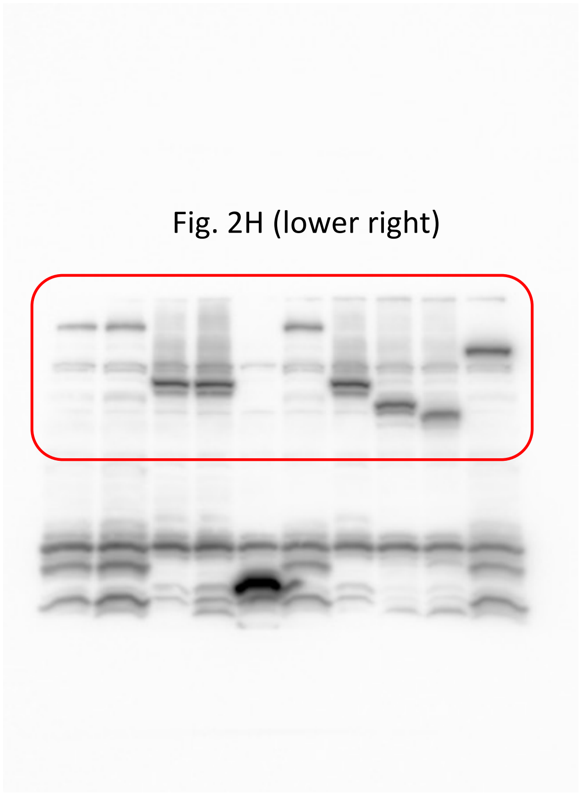

Journal of Cell Science • Supplementary information

Fig. S4G: Uncropped images of Fig. 3C

Fig. 3C (upper right) Fig. 3C (upper left)

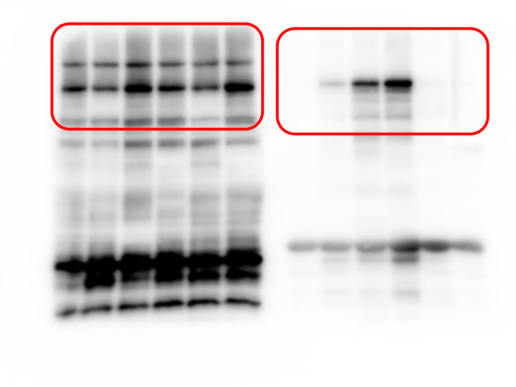

Fig. 3C (lower right) Fig. 3C (lower left)

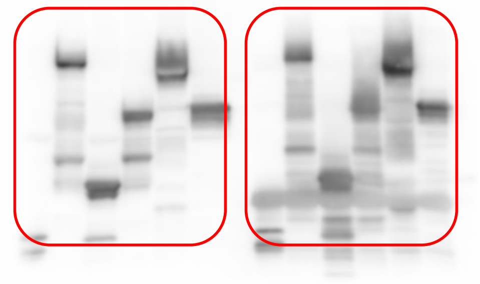

Journal of Cell Science • Supplementary information

Fig. S4H: Uncropped images of Fig. 3E

Fig. 3E (upper left)

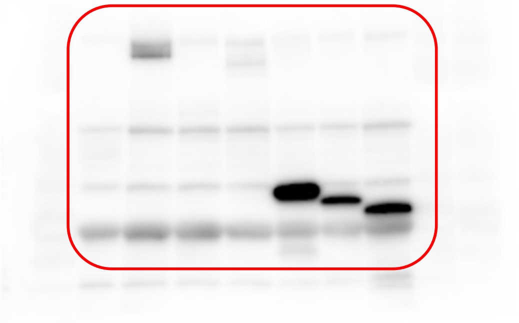

Fig. 3E (upper right)

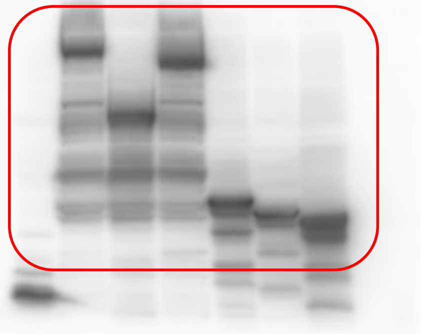

Fig. 3E (lower left)

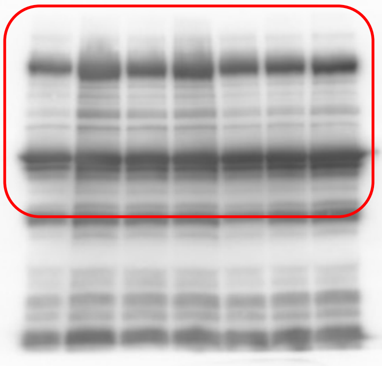

Fig. 3E (lower right)

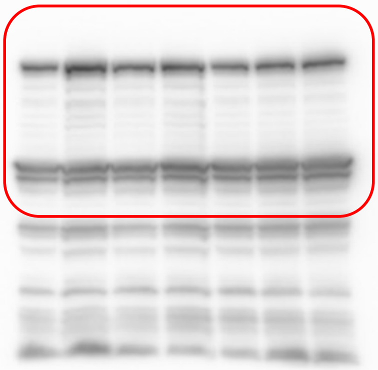

Journal of Cell Science • Supplementary information

Fig. S4I: Uncropped images of Fig. 3G

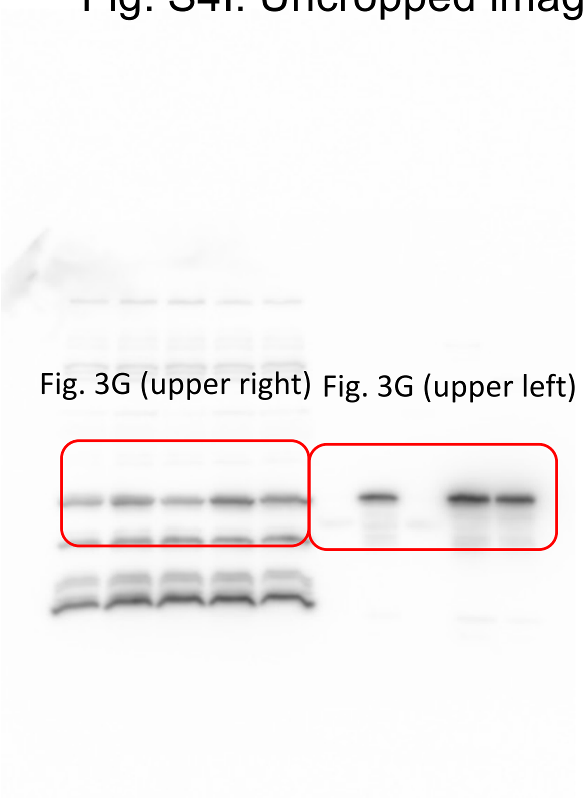

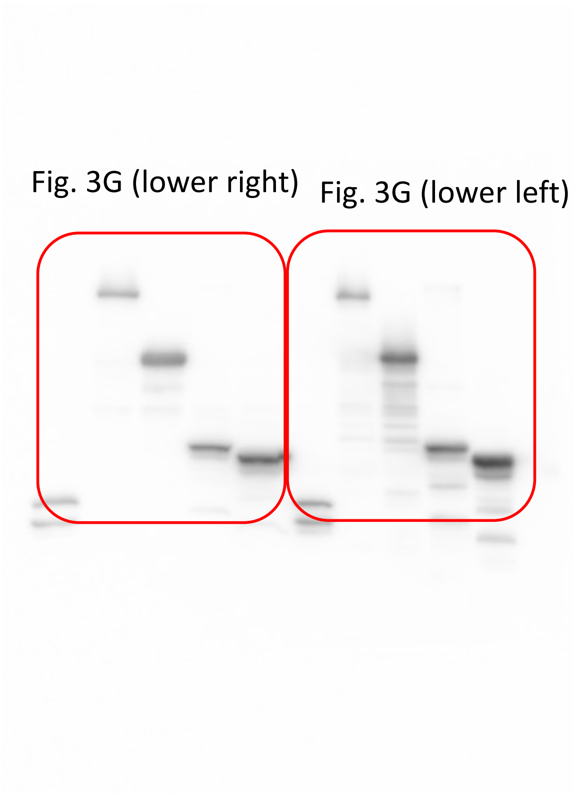

Journal of Cell Science • Supplementary information

Fig. S4J: Uncropped images of Fig. S1B

Fig. S1B (upper left)

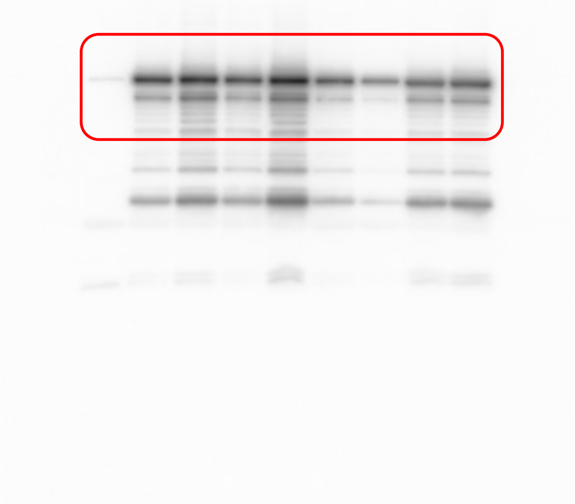

Fig. S1B (upper right)

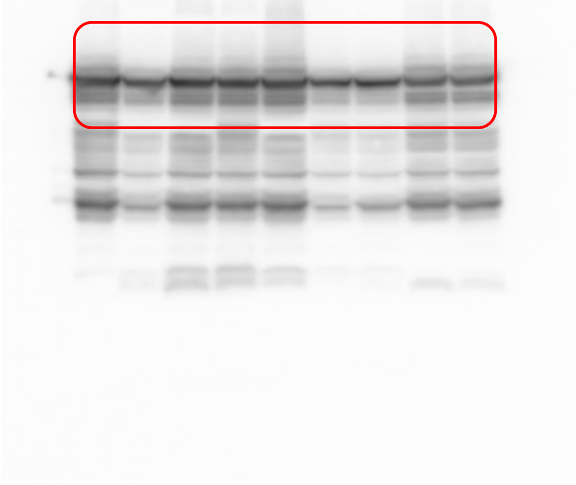

Fig. S1B (lower left)

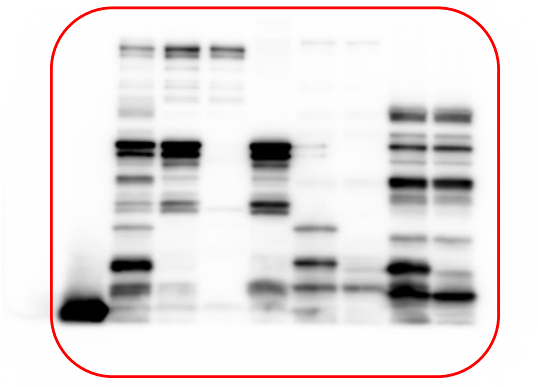

Fig. S1B (lower right)

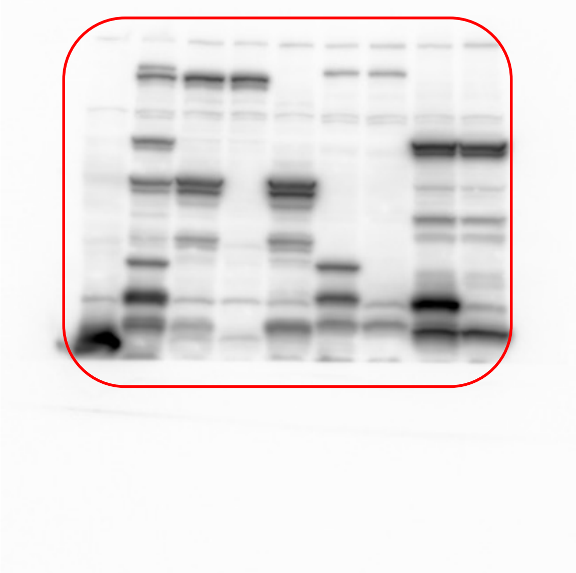

Journal of Cell Science • Supplementary information

Fig. S4K: Uncropped images of Fig. S1D

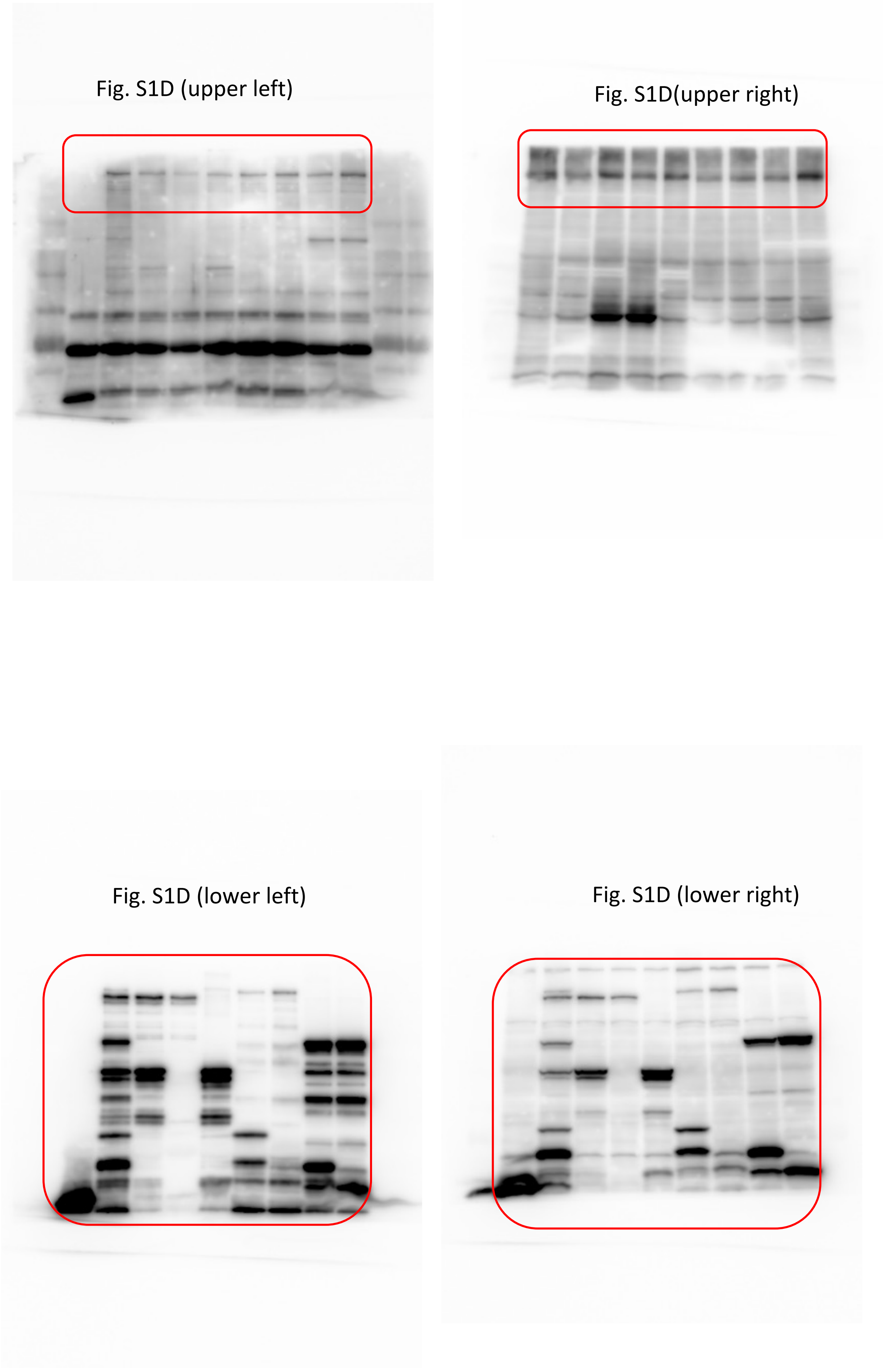

Journal of Cell Science • Supplementary information

Fig. S4L: Uncropped images of Fig. S1F

Fig. S1F (upper left)

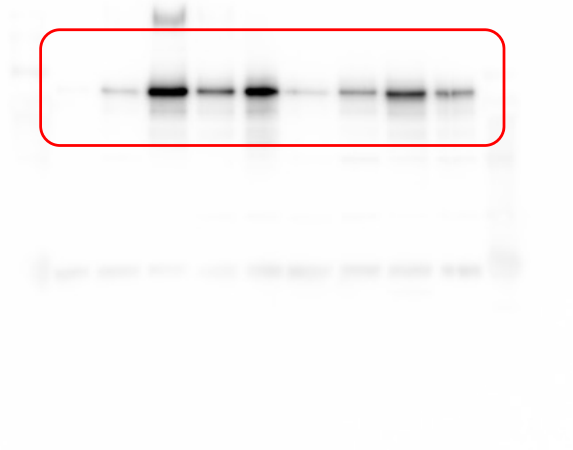

Fig. S1F(upper right)

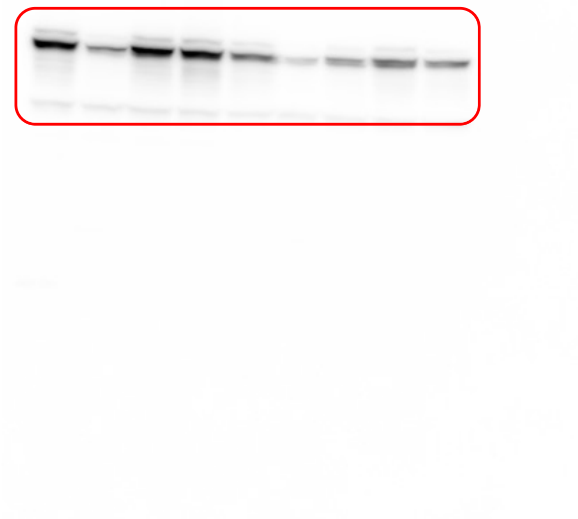

Fig. S1F (lower left)

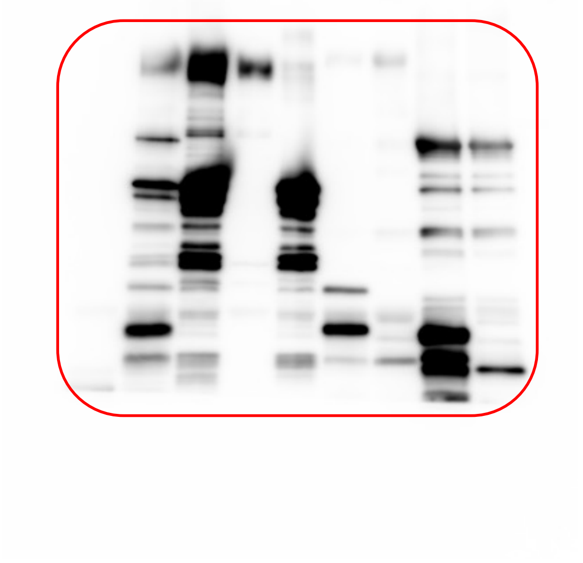

Fig. S1F (lower right)

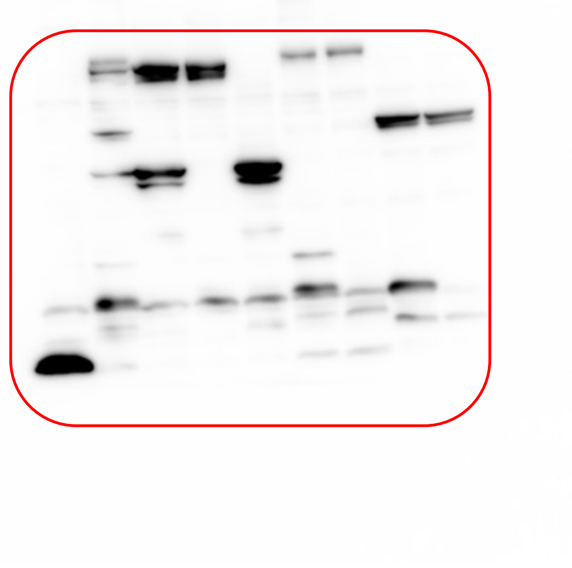

Journal of Cell Science • Supplementary information

Fig. S4M: Uncropped images of Fig. S1H

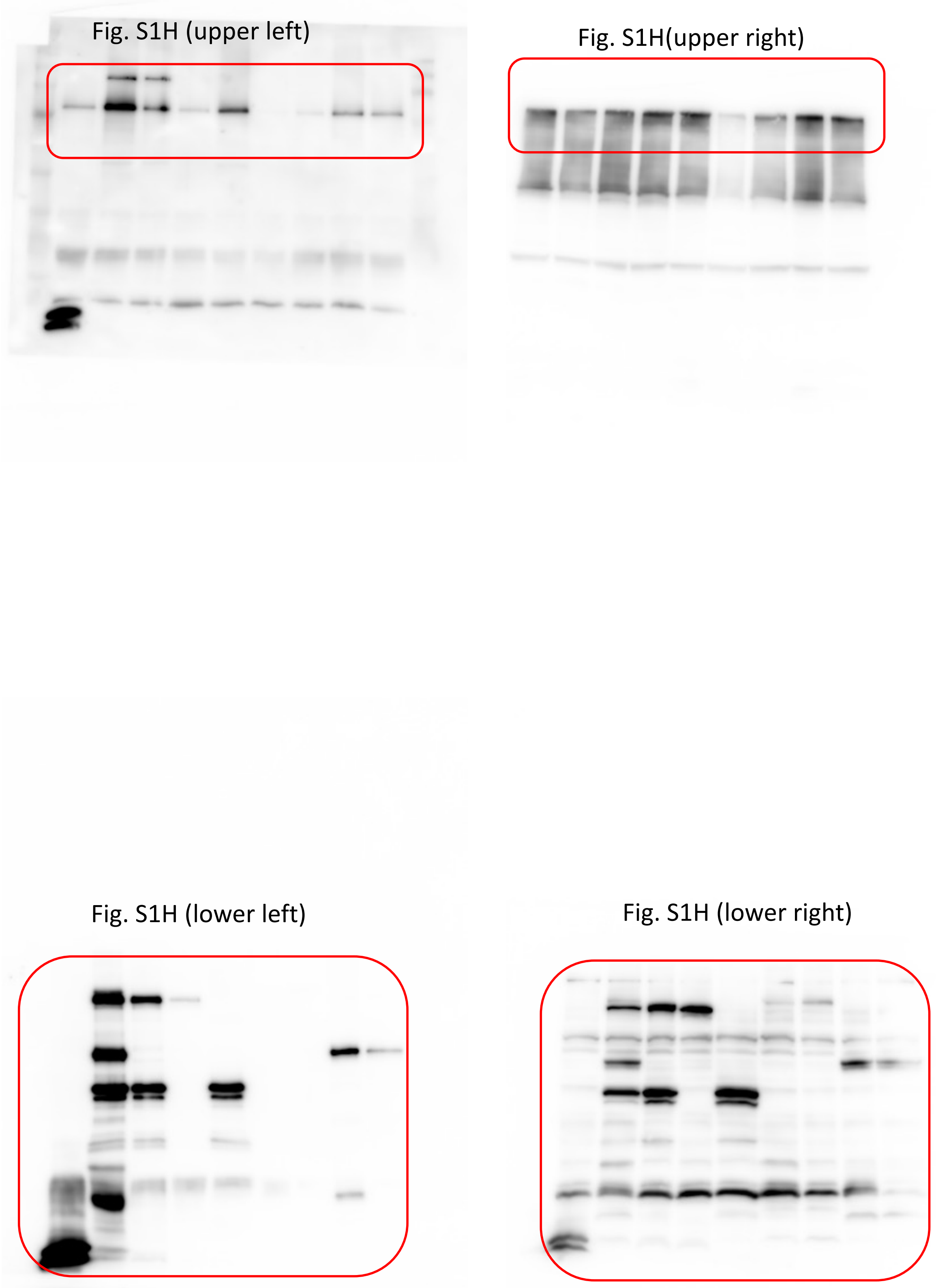

Journal of Cell Science • Supplementary information

Table S1. Plasmids used in this study

Vector	Insert *	Reference
pCAG2-EGFP-C	DYNC2LI1	Hamada et al., 2018
pCAG2-EGFP-N	DYNC2H1(N; 1–1,090)	Qiu et al., 2022
pCAG2- mCherry-C	WDR60(627–1,066)	Qiu et al., 2022
pCAG2- mCherry-C	IFT54	This study
pCAG2-EGFP-C	DYNC2LI1(1–240)	Qiu et al., 2022
pCAG2-EGFP-C	DYNC2LI1(241–352)	Qiu et al., 2022
pCAG2-EGFP-C	WDR34	Hamada et al., 2018
pCAG2-EGFP-C	WDR60	Hamada et al., 2018
pCAG2-EGFP-C	DYNLL1	Hamada et al., 2018
pCAG2-EGFP-C	DYNLL2	Hamada et al., 2018
pCAG2-EGFP-C	DYNLRB1	Hamada et al., 2018
pCAG2-EGFP-C	DYNLRB2	Hamada et al., 2018
pCAG2-EGFP-C	DYNLT1	Hamada et al., 2018
pCAG2-EGFP-C	DYNLT3	Hamada et al., 2018
pCAG2-EGFP-C	TCTEX1D2	Hamada et al., 2018
pCAG2-mCherry-C	DYNLL1	Hamada et al., 2018
pmCherry-C1	DYNLL2	Hamada et al., 2018
pCAG2-mCherry-C	DYNLRB1	Hamada et al., 2018
pCAG2-mCherry-C	DYNLRB2	Hamada et al., 2018
pCAG2-mCherry-C	DYNLT1	Hamada et al., 2018
pCAG2-mCherry-C	DYNLT3	Hamada et al., 2018
pCAG2-mCherry-C	TCTEX1D2	Hamada et al., 2018
pCAG2-EGFP-C	WDR34(1-146)	Tsurumi et al., 2019
pCAG2-EGFP-C	WDR34(147-536)	Tsurumi et al., 2019
pCAG2-EGFP-C	WDR60(1-374)	This study
pCAG2-EGFP-C	WDR60(1-626)	This study
pCAG2-EGFP-C	WDR60(375-1066)	Hamada et al., 2018
pCAG2-EGFP-C	WDR60(395-1066)	This study
pCAG2-EGFP-C	WDR60(473-1066)	Hamada et al., 2018
pCAG2-EGFP-C	WDR60(523-1066)	Hamada et al., 2018
pCAG2-EGFP-C	WDR60(574-1066)	This study
pCAG2-EGFP-C	WDR60(627-1066)	Qiu et al., 2022
pCAG2-EGFP-C	WDR60(Δ375–394)	This study
pEGFP-N	IFT20	Katoh et al., 2016
pmCherry-N	IFT20	Zhou et al., 2022
pmCherry-C1	IFT22	Zhou et al., 2022

Journal of Cell Science • Supplementary information

pmCherry-C1	IFT25	Zhou et al., 2022
pmCherry-C1	IFT27	Zhou et al., 2022
pCAG2-mCherry-C	IFT38	This study
pCAG-mCherry-C	IFT46	Katoh et al., 2016
pCAG-mCherry-C	IFT52	Katoh et al., 2016
pCAG2-mCherry-C	IFT56	This study
pCAG2-mCherry-C	IFT57	Katoh et al., 2016
pCAG-mCherry-C	IFT70	Katoh et al., 2016
pCAG-mCherry-C	IFT74	Katoh et al., 2016
pCAG-mCherry-N	IFT80	Katoh et al., 2016
pCAG-mCherry-C	IFT81	Katoh et al., 2016
pCAG-mCherry-C	IFT88	Katoh et al., 2016
pCAG-mCherry-C	IFT172	Katoh et al., 2016
pCAG2-mCherry-C	IFT54(1-134)	This study
pCAG2-mCherry-C	IFT54(1-334)	This study
pCAG2-mCherry-C	IFT54(335-625)	This study
pCAG2-mCherry-C	IFT54(488-625)	This study
pCAG2-mCherry-C	IFT54(507-625)	This study
pCAG2-mCherry-C	IFT54(533-625)	This study
pmCherry-C1	IFT43	Hirano et al., 2017
pCAG2-mCherry-C	IFT121	Hirano et al., 2017
pCAG2-mCherry-C	IFT122	Hirano et al., 2017
pCAG2-mCherry-C	IFT139	Hirano et al., 2017
pCAG2-mCherry-C	IFT140	Hirano et al., 2017
pCAG2-mCherry-C	IFT144	Hirano et al., 2017
pmCherry-C1	TULP3	Hirano et al., 2017
pRRLsinPPT-mCherry-C-IRES-Blast	WDR60	Hamada et al., 2018
pRRLsinPPT-mCherry-C-IRES-Blast	WDR60(1-626)	This study
pRRLsinPPT-mCherry-C-IRES-Blast	WDR60(375-1066)	This study
pRRLsinPPT-mCherry-C-IRES-Blast	WDR60(395-1066)	This study
pRRLsinPPT-mCherry-C-IRES-Blast	WDR60(Δ375-394)	This study
pRRLsinPPT-EGFP-C	DYNC2LI1	Hamada et al, 2018
pGEX-6P1	Anti-GFP-nanobody	Katoh et al., 2015
pGEX-6P1	Anti-mCherry-Nanobody (LaM-2)	Ishida et al., 2021

* All cDNA inserts except for those of anti-GFP and anti-mCherry Nbs are of human origin.

Journal of Cell Science • Supplementary information

Table S2. Antibodies used in this study

Antibody	Manufacturer	Clone/catalog number or reference number	Dilution (purpose)
Polyclonal rabbit anti-IFT88	Proteintech	13967-1-AP	1:500 (IF)
Polyclonal rabbit anti-IFT140	Proteintech	17460-1-AP	1:500 (IF)
Polyclonal rabbit anti-GPR161	Proteintech	13398-1-AP	1:200 (IF)
Polyclonal rabbit anti-ARL13B	Proteintech	17711-1-AP	1:500 (IF)
Monoclonal mouse anti-ARL13B	Abcam	N295B/66	1:500 (IF)
Monoclonal mouse anti-FOP	Abnova	2B1	1:10,000 (IF)
Monoclonal mouse anti-Smoothened	Santa Cruz	sc-166685	1:100 (IF)
Polyclonal goat anti-CEP164	Santa Cruz	sc-240226	1:500 (IF)
Monoclonal mouse anti-acetylated-α-tubulin	Sigma-Aldrich	6-11-B	1:2000 (IF)
Monoclonal mouse anti-RFP	MBL	3G5	1:1,000 (IF)
Polyclonal rabbit anti-mCherry	Proteintech	26765-1-AP	1:10,000 (IB)
Monoclonal mouse anti-GFP	Proteintech	66002-1-Ig	1:10,000 (IB)
AlexaFluor-conjugated secondary	Molecular Probes	A11034, A21127,-A21241, A21131, A21242, A21245	1:1,000 (IF)
Peroxidase-conjugated secondary	Jackson ImmunoResearch	115-035-166, 111-035-144	1:3,000 (IB)

IF, immunofluorescence; IB, immunoblotting

Journal of Cell Science • Supplementary information

Supplementary references

Hamada, Y., Tsurumi, Y., Nozaki, S., Katoh, Y., and Nakayama, K. (2018). Interaction of WDR60 intermediate chain with TCTEX1D2 light chain of the dynein-2 complex is crucial for ciliary protein trafficking. Mol. Biol. Cell *29*, 1628-1639.

Hirano, T., Katoh, Y., and Nakayama, K. (2017). Intraflagellar transport-A complex mediates ciliary entry and retrograde trafficking of ciliary G protein-coupled receptors. Mol. Biol. Cell *28*, 429-439.

Ishida, Y., Kobayashi, T., Chiba, S., Katoh, Y., and Nakayama, K. (2021). Molecular basis of ciliary defects caused by compound heterozygous *IFT144/WDR19* mutations found in cranioectodermal dysplasia. Hum. Mol. Genet. *30*, 213-225.

Katoh, Y., Terada, M., Nishijima, Y., Takei, R., Nozaki, S., Hamada, H., and Nakayama, K. (2016). Overall architecture of the intraflagellar transport (IFT)-B complex containing Cluap1/IFT38 as an essential component of the IFT-B peripheral subcomplex. J. Biol. Chem. *291*, 10962-10975.

Qiu, H., Tsurumi, Y., Katoh, Y., and Nakayama, K. (2022). Combinations of deletion and missense variations of the dynein-2 DYNC2LI1 subunit found in skeletal ciliopathies cause ciliary defects. Sci. Rep. *12*, 31.

Tsurumi, Y., Hamada, Y., Katoh, Y., and Nakayama, K. (2019). Interactions of the dynein-2 intermediate chain WDR34 with the light chains are required for ciliary retrograde protein trafficking. Mol. Biol. Cell *30*, 658-670.

Zhou, Z., Qiu, H., Castro-Araya, R.-F., Takei, R., Nakayama, K., and Katoh, Y. (2022). Impaired cooperation between IFT74/BBS22–IFT81 and IFT25–IFT27/BBS19 in the IFT-B complex causes ciliary defects in Bardet-Biedl syndrome. Hum. Mol. Genet. *31*, 1681-1693.

Journal of Cell Science • Supplementary information
