# Supplementary material for: Multiple interactions of the dynein-2 complex with the IFT-B complex are required for effective intraflagellar transport
Source: J Cell Sci. 2023 Feb 7;136(5):jcs260462. doi: 10.1242/jcs.260462 (PMC10110421; doi:10.1242/jcs.260462)
Supplement: Supplementary file 1 [file joces-136-260462_review_history.pdf]

Multiple interactions of the dynein-2 complex with the IFT-B complex are required for effective intraflagellar transport

Shunya Hiyamizu, Hantian Qiu, Laura Vuolo, Nicola L. Stevenson, Caroline Shak, Kate J. Heesom, Yuki Hamada, Yuta Tsurumi, Shuhei Chiba, Yohei Katoh, David J. Stephens and Kazuhisa Nakayama
DOI: 10.1242/jcs.260462

Editor: Guangshuo Ou

Review timeline

Original submission:	28 July 2022
Editorial decision:	30 August 2022
First revision received:	24 November 2022
Editorial decision:	28 December 2022
Second revision received:	29 December 2022
Accepted:	4 January 2023

Original submission

First decision letter

MS ID#: JOCES/2022/260462

MS TITLE: Multiple interactions of the dynein-2 complex with the IFT-B complex are required for effective IFT

AUTHORS: Shunya Hiyamizu, Hantian Qiu, Laura Vuolo, Nicola L. Stevenson, Caroline Shak, Kate J. Heesom, Yuki Hamada, Yuta Tsurumi, Shuhei Chiba, Yohei Katoh, David J. Stephens, and Kazuhisa Nakayama
ARTICLE TYPE: Research Article

We have now reached a decision on the above manuscript.

To see the reviewers' reports and a copy of this decision letter, please go to: https://submit-jcs.biologists.org and click on the 'Manuscripts with Decisions' queue in the Author Area. (Corresponding author only has access to reviews.)

As you will see, the reviewers raise a number of substantial criticisms that prevent me from accepting the paper at this stage. They suggest, however, that a revised version might be considered, if you can address their concerns. If you think that you can deal satisfactorily with the Reviewer #2's criticisms on revision, I would be pleased to see a revised manuscript. We would then return it to the reviewers.

Please ensure that you clearly highlight all changes made in the revised manuscript. Please avoid using 'Tracked changes' in Word files as these are lost in PDF conversion.

I should be grateful if you would also provide a point-by-point response detailing how you have dealt with the points raised by the reviewers in the 'Response to Reviewers' box. Please attend to all of the reviewers' comments. If you do not agree with any of their criticisms or suggestions please explain clearly why this is so.

© 2023. Published by The Company of Biologists under the terms of the Creative Commons Attribution License (https://creativecommons.org/licenses/by/4.0/).

Reviewer 1

Advance summary and potential significance to field

In this manuscript Hiyamizu et al. identify novel interactions between dynein-2 subunits and components of the IFT-B complex, identify protein domains involved and show that these interactions are important for proper IFT. These analyses are a further important step towards understanding how the IFT machinery works.

Comments for the author

Major comments
Statistics. In Figs 4 and 6 the authors use statistics to test whether deletion constructs show a significantly different effect than the WT construct. These are good comparisons. However, the authors never analyze whether wt RPE1 cells differ from WDR60 KO cells, or whether cells transfected with the WT construct differ from the KO or the WT cells. Please include these statistical analyses.

Fig 5A-D show very nice super resolution images of how IFT88 localization is affected by mutation of WDR60, WDR34 or DYNC2LI1. To be able to draw conclusions from these analyses the authors should quantify their results, preferably by scanning fluorescence intensities along the length of the cilium of many cilia and comparing the distribution of the fluorescence intensities between the different conditions.

Minor comments
In the introduction, the authors describe the composition of the IFT complexes. However, these are not the same in all organisms and the authors do not state which organism their description is based on. Please indicate this.

I got lost in the last sentence of the introduction. I understand what is meant, I think, and the authors are being careful not to claim too much, but the sentence is quite unclear. Please rephrase.

I found the text in the figures 1, 2 and 6, and S1 too small. Please increase the font size a bit. Figure 3 is fine.

There is a word missing in the sentence “whilst IFT172 was 6 of 7 WDR60 proteomes” half way the first paragraph of the results.

On page 6 and Fig 1C and D, the authors show that omitting IFT54 reduced fluorescence in the VIP assay. However, still many IFT-B proteins could be precipitated. How can this be? Please explain.

Fig 4. The authors look at differences in cilium length and length variability. This is fine, but it would be helpful to indicate in the fig (in addition to in the legend) that black lines address cilium length and green the variability.

Page 10, Fig 4H. The authors state that “In WDR60-KO cells expressing mCherry-fused WDR60(Δ375-395)…… the ciliary length variability (Fig. 4G, H) …… was not significantly altered compared with those expressing WDR60(WT).” However, Fig 4H indicates that $p<0.0001$. Please explain.

Page 10 states that IFT88 was predominantly found in the distal appendage region

Reviewer 2

Advance summary and potential significance to field

Trafficking of ciliary proteins mediated by the intraflagellar transport (IFT) is a fascinating and rapidly advancing subject. Import and export of ciliary protein cargoes must cross the diffusion barrier near the ciliary base, often facilitated by the IFT complexes. The anterograde and

© 2023. Published by The Company of Biologists under the terms of the Creative Commons Attribution License (https://creativecommons.org/licenses/by/4.0/).

retrograde movements mediated by IFT complexes, are driven by kinesin-II and dynein-2 motors respectively. As the IFT transport typically moves in one direction until reaching the ends of the axoneme, the anterograde and retrograde motor proteins must coordinate their activities to avoid the “tug of war” in transit. In particular, during anterograde IFT, the dynein-2 must remain inactive and act as a cargo until when the remodeling of the IFT complexes at the ciliary tip activates dynein-2 for retrograde IFT.
In this manuscript, Hiyamizu et al. built on previous discovery of IFT-B subunits co-precipitated with dynein intermediate chains WDR60 and/or WDR34, and sought to identify the interactions between IFT-B complexes and dynein-2. A recent study revealed human dynein-2 structure by cryo-EM (Toropova et al., 2019) suggested extensive interactions between inactive dynein-2 and the anterograde IFT train. In light of the suggested interactions, this study is particularly relevant and potentially informs the identification of such interactions at a sub-molecular or atomic level. Furthermore, this study may provide a basis of a “docking model” proposed by the author, where the anterograde transport dynein-2 relies on the extensive interactions with IFT-B rather than IFT-A or other subcomplexes. Revealing the structural and functional nature of the IFT-dynein-2 interactions would no doubt provide insight into interactions between large molecular complexes and broaden the understanding of the cilia-related diseases.

Comments for the author

For transparency, the decision letter from Dr. Gregory Pzaour serving as monitoring editor for MBoC was included in the reviewer package, along with authors’ rebuttal to the last round of comments from two previous reviewers.

As the authors pointed out, the interaction profiled in this study may provide the first experimental evidence to support the proposed interaction between dynein 2 and IFT-B in Toropova et al., 2019 study. In my view, the interactions documented in this manuscript are limited in scope and detail to sufficiently validate the proposed interactions during anterograde IFT. The investigation and interpretation of the function aspects of the interactions concerning cilia length, IFT-B entry and distribution and HH signaling, are also inadequate.
Therefore, it is my opinion that the present manuscript doesn’t provide sufficient contribution to the understanding of dynein-2 and IFT interaction nor does it provide adequate evidence for mechanistic insight, therefore is unsuitable for publication in JCS.

Major limitations:
1. The “docking model” was highlighted several times and was clearly the intended hypothesis/conclusion. However, as the author acknowledged the limitation of the presented data in supporting such a claim, the omission of alternative and more intuitive models is misleading for readers unfamiliar with the subject. For example, dynein 2 is more likely to form robust interactions with IFT machinery during retrograde transport that are consistent with its active state. The proteomic data and VIP assay do not differentiate the interactions on the anterograde versus the retrograde trains. Furthermore, can the functional results be explained by an alternative model where WDR60-IFT54 interaction is present at retrograde IFT? Related to this, previous reviewer 1 pointed out the tip enrichment of IFT88 in various truncations consistent with a retrograde IFT defect. It’s unclear what the authors’ response is.

2. The modification made in response to previous reviewer 1’s comment about “interactions present in the anterograde IFT” was certainly noticeable. With these changes, the manuscript still lacked evidence that would advance the understanding of these interactions at a mechanistic level. While the proteomic and VIP assay dataset are robust and mostly consistent, they only provide limited information. For example, do other dynein-2 components also primarily interact with IFT-B subunits? Other than the most robust IFT54-WDR60 interaction, what are the other pairings? While a comprehensive mapping of the interactions between IFT subunits and dynein 2 subunits is likely too ambitious further investigation into some of the less robust interactions is a sensible goal in order to experimentally validate the claim about the extended dynein 2-IFT-B interactions.

3. The LC-MS/MS data summarized in Table 1 potentially directly supports a robust interaction between dynein 2 and IFT-B. Though the analysis shown must be expanded more adequately for evaluation. For example, what was the experimental baseline for the >2 fold enrichment? Was the

© 2023. Published by The Company of Biologists under the terms of the Creative Commons Attribution License (https://creativecommons.org/licenses/by/4.0/).

fold changes calculated based on an average of all peptides detected? How was the coverage of the identified subunits? Why was 2 fold chosen as the threshold? Please note PRIDE confirmed the proteomics data was submitted, but this reviewer did not find the necessary credential to access them, hence can not evaluate the authors' claims or potential drawbacks.

4. What did the HH signaling analysis tell about the interactions? A conclusion from these data was not suggested and the observed defects did not correlate with the ciliary length or IFT88 analysis. This result was largely ignored in the abstract and only mentioned trivially in discussion. The authors might consider giving a brief introduction about the role of dynein 2 in ciliary entry and exit, and provide their interpretation that readers can assess.

As the data reported is consistent with previous findings and may lead to significant progress. A resubmission should be considered. Based on my interest in this study, some of my immature suggestions for resubmission are (a) classifying the interaction(s) by experimentally examining anterograde IFT trains and retrograde IFT trains such as using EM/Cryo-EM and developing the "docking model" further, (b) expanding the effort to map additional interactions between dynein-2 and IFT subunits, (c) revelation of one or several interactions at a mechanistic level or at a particular step of IFT.

Minor comments:
1. It appears the Δ375-395 version of WDR60 enriched as faint dots at the base of cilia (mChe) in Fig. 4O, 6G, 6U and 6BB but not in 6N, while others were consistently undetectable in cilia. WDR60 Δ375-395 was not highly expressed in Fig. 2 D. Can the authors comment on the ciliary base enrichment?

2. In Fig. 4, WDR60 Δ375-395 rescued the IFT88 distribution phenotype of WDR60 KO while other trunctions did not. Does this also apply to IFT140 and IFT43 distribution phenotypes reported in Vuolo et al., 2018. Because the cilia length and IFT88 distribution data are inconclusive and the phenotypes can't be simply explained by the lack of 375-395 region, therefore how retrograde IFT components were affected in these conditions may give a more complete picture of the defect.

3. Cilia length variability of WDR60 Δ375-395 in WDR60-KO was described as "not significantly altered" (page 10, paragraph 2, line 6) compared to WT which contradicted with Fig. 4H top green horizontal bar.

4. Reference to "Fig. S2" at page 13, paragraph 2, line 15 should be "Fig. S1".

First revision

Author response to reviewers' comments

Our response to the reviewers' comments

Reviewer 1 Comments for the author

Major comments

Statistics. In Figs 4 and 6 the authors use statistics to test whether deletion constructs show a significantly different effect than the WT construct. These are good comparisons. However, the authors never analyze whether wt RPE1 cells differ from WDR60 KO cells, or whether cells transfected with the WT construct differ from the KO or the WT cells. Please include these statistical analyses.

For the data in Figs. 4 and 6, we did perform statistical analyses between control RPE1 cells and *WDR60*-KO cells or those expressing various constructs. However, in the previous version, we intentionally omitted these comparisons because we thought they would crowd the graph. We have therefore added the results of statistical analyses in the revised versions of Fig. 4H, 4P, 6CC, or 6DD.

© 2023. Published by The Company of Biologists under the terms of the Creative Commons Attribution License (https://creativecommons.org/licenses/by/4.0/).

Fig 5A-D show very nice super resolution images of how IFT88 localization is affected by mutation of WDR60, WDR34 or DYNC2LI1. To be able to draw conclusions from these analyses the authors should quantify their results, preferably by scanning fluorescence intensities along the length of the cilium of many cilia and comparing the distribution of the fluorescence intensities between the different conditions.

According to the reviewer's suggestion, we have performed line-scanning of IFT88 staining intensities along individual cilia, added the scanning data (new Fig. 5, E-H), and modified the corresponding paragraph (page 11, the second paragraph (the bottom paragraph in the converted PDF); highlighted in green). However, in this case, the scans were performed on IFT88 stained images acquired by conventional microscopy for a technical reason.

Minor comments

In the introduction, the authors describe the composition of the IFT complexes. However, these are not the same in all organisms and the authors do not state which organism their description is based on. Please indicate this.

In INTRODUCTION (pages 3 and 4; highlighted in green), we have tried to mention as much as possible about the species of the IFT components.

I got lost in the last sentence of the introduction. I understand what is meant, I think, and the authors are being careful not to claim too much, but the sentence is quite unclear. Please rephrase.

According to the reviewer's point, we have removed "with an interaction between WDR60 and IFT54 being of particular importance" in the last sentence of INTRDCTION.

I found the text in the figures 1, 2 and 6, and S1 too small. Please increase the font size a bit. Figure 3 is fine.

For Figs. 1, 2, 6, and S1, the font size has been slightly increased.

There is a word missing in the sentence "whilst IFT172 was 6 of 7 WDR60 proteomes" half way the first paragraph of the results.

"whilst IFT172 was 6 of 7 WDR60 proteomes" has been changed to "whilst IFT172 was found in 6 of 7 WDR60 proteomes".

On page 6 and Fig 1C and D, the authors show that omitting IFT54 reduced fluorescence in the VIP assay. However, still many IFT-B proteins could be precipitated. How can this be? Please explain.

Although we do not know the exact reason for the apparent difference between the subtractive VIP and immunoblotting data, we suspect that despite the reduction in the red fluorescence when mCherry-IFT54 was omitted, the other subunits retained their abilities to bind to the dynein-2 complex, albeit somewhat weakly (A new statement was added in the text; page 6, the second paragraph; highlighted in green).

Fig 4. The authors look at differences in cilium length and length variability. This is fine, but it would be helpful to indicate in the fig (in addition to in the legend) that black lines address cilium length and green the variability.

In addition to the Fig. 4H legend, we have described in the main text what the black lines and letters and the green lines and letters mean (page 10, the second paragraph; highlighted in green).

Page 10, Fig 4H. The authors state that "In WDR60-KO cells expressing mCherry-fused WDR60(Δ375–394)…… the ciliary length variability (Fig. 4G, H) …… was not significantly altered compared with those expressing WDR60(WT)." However, Fig 4H indicates that $p<0.0001$. Please explain.

© 2023. Published by The Company of Biologists under the terms of the Creative Commons Attribution License (https://creativecommons.org/licenses/by/4.0/).

Page 10 states that IFT88 was predominantly found in the distal appendage region.

This comment is essentially the same as the minor comment 3 of Reviewer #2. This description that the reviewers pointed out was completely our mistake. In fact, with respect to cilia length variation, there was a significant difference between WDR60-KO cells expressing WDR60(WT) and those expressing WDR60(Δ375-394). We have therefore corrected the corresponding statement (page 10, the bottom paragraph (page 11, the first paragraph in the converted PDF), highlighted in green) and modified the statement related to it in DISCUSSION (page 15 (page 16 in the converted PDF), the second paragraph, highlighted in green).

Reviewer 2 Comments for the Author:

Major limitations:

1. The "docking model" was highlighted several times and was clearly the intended hypothesis/conclusion. However, as the author acknowledged the limitation of the presented data in supporting such a claim, the omission of alternative and more intuitive models is misleading for readers unfamiliar with the subject. For example, dynein 2 is more likely to form robust interactions with IFT machinery during retrograde transport that are consistent with its active state. The proteomic data and VIP assay do not differentiate the interactions on the anterograde versus the retrograde trains. Furthermore, can the functional results be explained by an alternative model where WDR60-IFT54 interaction is present at retrograde IFT? Related to this, previous reviewer 1 pointed out the tip enrichment of IFT88 in various truncations consistent with a retrograde IFT defect. It's unclear what the authors' response is.

We agree with the point made by the reviewer. We cannot strictly distinguish whether the interactions between subunits of dynein-2 and IFT-B2, including the WDR60-IFT54 interactions, are important for the anterograde trafficking of dynein-2 or the dynein-2-driven retrograde trafficking.

However, as described in the cover letter to Prof. Ou, in the molecular model (NOT a docking model) of the *Chlamydomonas* anterograde IFT train assembled on the basis of the cryo-ET analysis and predictions using Alphafold2 (Pigino lab, Lacey et al., 2022, bioRxiv), the dynein-2 complex have multiple contacts with the IFT-B2 side of the IFT-B repeats. The interaction of the dynein-2 complex with the IFT-B2 side is also supported by the AlphaFold model of the IFT-B complex validated using cross-linking/MS data (Lorentzen lab, Petriman et al., 2022, EMBO J.). Thus, the molecular model of the anterograde IFT train is consistent with our data that the subunits of dynein-2, including WDR60 and DYNC2H1-DYNC2LI1, interact primarily with the IFT-B2 subunits. By citing this bioRxiv paper, we have therefore enhanced our discussion about the role of the interaction between dynein-2 and IFT-B2 (highlighted in yellow in the revised manuscript).

Nevertheless, we have added the following statement to emphasize that we cannot strictly distinguish whether the interactions between subunits of dynein-2 and IFT-B2 are important for the anterograde trafficking of dynein-2 or the dynein-2-driven retrograde trafficking.

(page 13 (page 14 in the converted PDF), the first paragraph; highlighted in blue) the interactions of WDR60, WDR34, and DYNC2H1-DYNC2LI1 with multiple IFT-B subunits are likely to mainly represent those occurring when the dynein-2 complex is transported as an anterograde IFT cargo, although the possibility that these interactions also occur during dynein-2-driven retrograde trafficking cannot be completely excluded.

2. The modification made in response to previous reviewer 1's comment about "interactions present in the anterograde IFT" was certainly noticeable. With these changes, the manuscript still lacked evidence that would advance the understanding of these interactions at a mechanistic level. While the proteomic and VIP assay dataset are robust and mostly consistent, they only provide limited information. For example, do other dynein-2 components also primarily interact with IFT-B subunits? Other than the most robust IFT54- WDR60 interaction, what are the other pairings? While a comprehensive mapping of the interactions between IFT subunits and dynein 2 subunits is likely too ambitious, further investigation into some of the less robust interactions is a sensible goal in order to experimentally validate the claim about the extended dynein 2-IFT- B interactions.

See below (the response to the reviewer's suggestion (b)).

© 2023. Published by The Company of Biologists under the terms of the Creative Commons Attribution License (https://creativecommons.org/licenses/by/4.0/).

3. The LC-MS/MS data summarized in Table 1 potentially directly supports a robust interaction between dynein 2 and IFT-B. Though the analysis shown must be expanded more adequately for evaluation. For example, what was the experimental baseline for the >2 fold enrichment? Was the fold changes calculated based on an average of all peptides detected? How was the coverage of the identified subunits? Why was 2 fold chosen as the threshold? Please note PRIDE confirmed the proteomics data was submitted, but this reviewer did not find the necessary credential to access them, hence can not evaluate the authors' claims or potential drawbacks.

In MATRIALS AND METHODS, we have described in detail the methodology of proteomic data analysis (the section spanning pages 17 and 18 (pages 18 and 19 in the converted PDF); highlighted in blue).

4. What did the HH signaling analysis tell about the interactions? A conclusion from these data was not suggested and the observed defects did not correlate with the ciliary length or IFT88 analysis. This result was largely ignored in the abstract and only mentioned trivially in discussion. The authors might consider giving a brief introduction about the role of dynein 2 in ciliary entry and exit, and provide their interpretation that readers can assess.

As the reviewer pointed out, there was a lack of description regarding the role of dynein-2 in retrograde trafficking/export of GPR161 and SMO and thereby in the regulation of the Hh signaling. We have modified the relevant text to clarify the role of dynein-2 in retrograde trafficking/export of GPR161 and SMO (highlighted in blue: INTRODUCTION, page 3, the second paragraph; RESULTS, page 12, the second paragraph; DISCUSSION, page 16 (page 17 in the converted PDF), the bottom paragraph).

As the data reported is consistent with previous findings and may lead to significant progress. A resubmission should be considered. Based on my interest in this study, some of my immature suggestions for resubmission are (a) classifying the interaction(s) by experimentally examining anterograde IFT trains and retrograde IFT trains such as using EM/Cryo-EM and developing the "docking model" further, (b) expanding the effort to map additional interactions between dynein-2 and IFT subunits, (c) revelation of one or several interactions at a mechanistic level or at a particular step of IFT.

As described in the cover letter to Prof. Ou, we have selected the reviewer's suggestion (b) and accordingly performed additional experiments to expand the interaction map between the dynein-2 and IFT-B subunits. Specifically, for IFT38 and IFT80, we have also examined their interaction with the dynein-2 subunits (new Fig. S1, E-H), in addition to IFT57 and IFT172 (Fig. S1, A-D), and consequently modified the corresponding paragraph (page 7, the second paragraph). The results indicate that IFT38 and IFT80 as well as IFT57 and IFT172 interact with multiple dynein-2 subunits. As mentioned many times in the text and as suggested by structural studies on the IFT train, the interactions between multiple IFT-B2 subunits and multiple dynein-2 subunits are so extensive and diverse that it is substantially difficult to refine the interaction map any further.

Minor comments:

1. It appears the Δ375-394 version of WDR60 enriched as faint dots at the base of cilia (mChe) in Fig. 4O, 6G, 6U and 6BB but not in 6N, while others were consistently undetectable in cilia. WDR60 Δ375-394 was not highly expressed in Fig. 2 D. Can the authors comment on the ciliary base enrichment?

Many thanks for the valuable comment. This comment has made us realize for the first time that mChe-WDR60(Δ375-394) tends to be enriched around the base of cilia. In Fig. 4, C-G, we have added enlarged red channel images as the rightmost panels. Among the three enlarged images in Fig. 4G, faint mCherry staining was found near the ciliary base in the bottom two images, like in Fig. 4O, 6G, 6U and 6BB. However, the background was relatively high because mCherry-WDR60 was detected by staining with anti- RFP antibodies due to the originally weak signals of mCherry-WDR60 constructs and the background depended on the individual mCherry-WDR60 constructs expressed. The high and variable background hindered our subsequent analysis. Therefore, in the revised MS, we have only described the fact that mChe- WDR60 (Δ375-394) tended to be enriched near the base of cilia (page 10, the bottom paragraph (page 11, the first paragraph in the converted PDF), highlighted in blue).

© 2023. Published by The Company of Biologists under the terms of the Creative Commons Attribution License (https://creativecommons.org/licenses/by/4.0/).

2. In Fig. 4, WDR60 Δ375-394 rescued the IFT88 distribution phenotype of WDR60 KO while other truncations did not. Does this also apply to IFT140 and IFT43 distribution phenotypes reported in Vuolo et al., 2018. Because the cilia length and IFT88 distribution data are inconclusive and the phenotypes can't be simply explained by the lack of 375-394 region, therefore how retrograde IFT components were affected in these conditions may give a more complete picture of the defect.

We had also examined IFT140 localization. However, we did not describe the IFT140 data in the previous version, as the IFT140 localization data were more inconclusive than the IFT88 data. Namely, IFT140 was significantly accumulated within cilia in WDR60-KO cells and those expressing WDR60(1-626), but we could not detect the IFT140 localization difference among WDR60-KO cells expressing WDR60(WT), WDR60(375-1066), WDR60(395-1066), and WDR60(Δ375-394). In the revised MS, we have added the IFT140 data as new Fig. S3 and added a new paragraph (page 11, the first paragraph (the second paragraph in the converted PDF), highlighted in blue).

3. Cilia length variability of WDR60 Δ375-394 in WDR60-KO was described as "not significantly altered" (page 10, paragraph 2, line 6) compared to WT which contradicted with Fig. 4H top green horizontal bar.

This comment is essentially the same as the last comment of Reviewer #1. According to the comment, we have corrected the corresponding statement (page 10, the bottom paragraph (page 11, the first paragraph in the converted PDF), highlighted in green).

4. Reference to "Fig. S2" at page 13, paragraph 2, line 15 should be "Fig. S1".

The (Fig. S2) has been corrected to (Fig. S1). (page 14 (page 15 in the converted PDF), the first paragraph in the revised MS)

Second decision letter

MS ID#: JOCES/2022/260462

MS TITLE: Multiple interactions of the dynein-2 complex with the IFT-B complex are required for effective IFT

AUTHORS: Shunya Hiyamizu, Hantian Qiu, Laura Vuolo, Nicola L. Stevenson, Caroline Shak, Kate J. Heesom, Yuki Hamada, Yuta Tsurumi, Shuhei Chiba, Yohei Katoh, David J. Stephens, and Kazuhisa Nakayama
ARTICLE TYPE: Research Article

We have now reached a decision on the above manuscript.

To see the reviewers' reports and a copy of this decision letter, please go to: https://submit-jcs.biologists.org and click on the 'Manuscripts with Decisions' queue in the Author Area. (Corresponding author only has access to reviews.)

As you will see, the reviewer gave a favourable report but raised an issue about citation that will require amendments to your manuscript. I hope that you will be able to carry this out because I would like to be able to accept your paper.

Please ensure that you clearly highlight all changes made in the revised manuscript. Please avoid using 'Tracked changes' in Word files as these are lost in PDF conversion.

I should be grateful if you would also provide a point-by-point response detailing how you have dealt with the points raised by the reviewers in the 'Response to Reviewers' box. Please attend to

© 2023. Published by The Company of Biologists under the terms of the Creative Commons Attribution License (https://creativecommons.org/licenses/by/4.0/).

all of the reviewers' comments. If you do not agree with any of their criticisms or suggestions please explain clearly why this is so.

Reviewer 2

Advance summary and potential significance to field

My summary of the previous version of this paper as reviewer #2, completed at 08/26/2022 for JCS, still mostly applies. Below is my summary specific to this revision:

In this revision, Hiyamizu et al. highlighted several most recently published studies and described how these studies and the current revision complement each other and provide structural and biochemical insights on the extensive interactions between dynein-2 complex and IFT-B complex. Additionally, this revision expanded the analysis and discussion regarding these interactions and others to provide the readers a comprehensive and balanced view. Despite certain technical difficulties and inconclusive results mentioned by the authors, the revised manuscript would make a significant contribution to this important subject. The authors made commendable effort to address every reviewers' comment by several additional experiments and made notable improvement on the scope and depth of this manuscript. In my opinion, this revision is therefore acceptable for publication in JCS.

Comments for the author

“Lacey et al., 2022” was cited several times throughout the revision and provided import support at several places throughout the manuscript. As this preprint is not peer-reviewed yet, the support provided should be considered carefully by the readers. Based on JCS Manuscript preparation policy 3.3.3.1, this reference should be cited as “(Lacey et al., 2022 preprint)” at all places including the Reference list. Please also seek editor’s advice.

Second revision

Author response to reviewers' comments

Reviewer 2 Comments for the author

“Lacey et al., 2022” was cited several times throughout the revision and provided. import support at several places throughout the manuscript. As this preprint is not peer-reviewed yet, the support provided should be considered carefully by the readers. Based on JCS Manuscript preparation policy 3.3.3.1, this reference should be cited as “(Lacey et al., 2022 preprint)” at all places including the Reference list. Please also seek editor’s advice.

Our response to the reviewer’s comment
We are sorry about not following the JCS manuscript preparation policy. According to the reviewer`s point, we have changed all “Lacey et al., 2022” to “Lacey et al., 2022 preprint”.

© 2023. Published by The Company of Biologists under the terms of the Creative Commons Attribution License (https://creativecommons.org/licenses/by/4.0/).

Third decision letter

MS ID#: JOCES/2022/260462

MS TITLE: Multiple interactions of the dynein-2 complex with the IFT-B complex are required for effective IFT

AUTHORS: Shunya Hiyamizu, Hantian Qiu, Laura Vuolo, Nicola L. Stevenson, Caroline Shak, Kate J. Heesom, Yuki Hamada, Yuta Tsurumi, Shuhei Chiba, Yohei Katoh, David J. Stephens, and Kazuhisa Nakayama
ARTICLE TYPE: Research Article

I am happy to tell you that your manuscript has been accepted for publication in Journal of Cell Science, pending standard ethics checks.

© 2023. Published by The Company of Biologists under the terms of the Creative Commons Attribution License (https://creativecommons.org/licenses/by/4.0/).
